# Correction: Persistence of Neighborhood Demographic Influences over Long Phylogenetic Distances May Help Drive Post-Speciation Adaptation in Tropical Forests

**DOI:** 10.1371/journal.pone.0168976

**Published:** 2016-12-20

**Authors:** Christopher Wills, Kyle E. Harms, Thorsten Wiegand, Ruwan Punchi-Manage, Gregory S. Gilbert, David Erickson, W. John Kress, Stephen P. Hubbell, C. V. Savitri Gunatilleke, I. A. U. Nimal Gunatilleke

## Notice of Republication

This article was republished on November 4, 2016, to correct errors in the figure order. Please download this article again to view the correct version. The originally published, uncorrected article and the republished, corrected articles are provided here for reference.

## Supporting Information

S1 File Originally published, uncorrected article. (PDF)

S2 File Republished, corrected article. (PDF)
