# Supplementary material for: Persistence of Neighborhood Demographic Influences over Long Phylogenetic Distances May Help Drive Post-Speciation Adaptation in Tropical Forests
Source: PLoS One. 2016 Jun 15;11(6):e0156913. doi: 10.1371/journal.pone.0156913 (PMC4909291; doi:10.1371/journal.pone.0156913)
Supplement: S1 File — (DOCX) [file pone.0156913.s001.docx]

**Supporting Information**

**Table of Contents Page**

**Detailed Methods 3**

**Text A. Details of the EAA method 3**

**Text B. Details of the subdivision of the FDP data sets 10**

**Fig. A: Growth values for smaller focal trees in the presence 12**

**of larger annular trees, for all annular tree basal areas and all phylogenetic**

**distances**

**Fig B. Sinharaja: Growth values for smaller focal trees in the presence 13**

**of larger annular trees, for all annular tree basal areas and all phylogenetic**

**distances**

**Fig. C. BCI: Growth values for larger focal trees in the presence 14**

**of smaller annular trees, for all annular tree basal areas and all phylogenetic**

**distances**

**Fig. D. Sinharaja: Growth values for larger focal trees in the presence 15**

**of smaller annular trees, for all annular tree basal areas and all phylogenetic**

**distances**

**Fig. E. BCI: Significances, presented as t-values and z-values, of the associations between focal trees of different size classes and their growth rates, and between their sizes and the degree of clustering, recruitment and mortality of their annular conspecifics, in all six subdivisions of the conspecific BCI data.**

**Fig. F. Sinharaja: Significances, presented as t-values and z-values, of the associations between focal trees of different size classes and their growth rates, and between their sizes and the degree of clustering, recruitment and mortality of their annular conspecifics, in all six subdivisions of the conspecific Sinharaja data.**

**Supporting information references 19**

**Detailed Methods**

**Text A. Details of the EAA Method**

In this section we give details of the EAA method, including the specific analyses using this method for the relationships between:

(1) focal tree growth rate and the presence or absence of different summed basal areas of conspecific or phylogenetically related annular trees,

(2) focal tree size and rates of conspecific or related heterospecific recruitment,

(3) focal tree size and rates of conspecific or related heterospecific mortality, and

(4) focal tree size and degree of clustering of the conspecific or phylogenetically related annular trees.

**(1) Analysis of influence of annular summed basal areas on focal tree growth rates.** In these analyses we examine differences in mean normalized growth rates of focal trees that are associated with (*1*) the presence or absence of specified summed basal areas of nearby annular conspecifics or (*2*) the presence or absence of specified summed basal areas of heterospecifics that lie within a given phylogenetic interval *P* from a focal tree. Normalized growth rates of focal trees are calculated by dividing the trees into ten dbh classes *d*, delimited by diameters of 10, 30, 50, 70, 90, 110, 130, 150, 170, 190, and >190 mm. Each tree’s growth rate *G* is expressed as the number of positive or negative standard deviations by which its growth rate deviates from the mean growth rate of its size class *d* within its species. For each annulus at a distance *r* from a focal tree, if conspecifics or heterospecifics in a given *P* from a focal tree are present, their summed basal areas *BC(r)* and *BP(r)* are distributed into different basal area categories.

Two classes of growth-rate analysis are presented here. The first measures the difference in the mean growth rate of small focal trees associated with the presence or absence of larger annular conspecific or phylogenetically related trees. In these analyses focal trees are examined that fall within the smallest diameter category, 10-30 mm (a maximum basal area of 700 mm2). For these small focal trees, annular tree basal area categories are delimited by values (in mm2) of 700, 950, 1,500, 3,000 and 10,000. Thus, all the annular tree categories examined have basal areas greater than that of the focal tree.

The second class of analyses measures the difference in the growth rate of the largest focal trees in the presence or absence of a smaller basal area of annular conspoecific or phylogenetically related trees. For large focal trees with a basal area of >= 28,000, annular tree basal area categories are delimited by values of 170, 2,000 and 28,000. In these analyses, annular tree basal areas are chosen that sum to less than the basal area of the focal tree.

For each annular distance *r*, the growth rates of focal trees that have conspecific or heterospecific annular trees that fall within phylogenetic interval *P,* and with summed basal areas falling within one of these size categories, are compared with the growth rates of focal trees that do not have conspecific trees or this category of heterospecific annular trees at this annular distance.

Focal trees are pooled over all census periods, on the assumption that their normalized growth rates can be treated as independent observations (see section on autocorrelations for a justification of this assumption).

In conspecific analyses the normalized growth rates of the focal trees are named GC*i,j,k*(*r,b*), where the arguments *r* and *b* indicate that we explicitly separate the effects of different basal area loads *b* of conspecific annular trees at different distances *r*. The mean growth rate of small focal trees with a basal area load *b* of conspecifics at annular distance *r* is then given by:


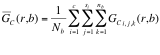
 , (1)

which is averaged over all individuals *k* of each species *j* and each census period *i*. The total number of census periods is *c*, the total number of focal species present during a census period *i* is *si*, the total number of focal trees of species *j* at census period *i* with basal area load *b* at distance *r* is *nb*, and *Nb* is the total number of focal trees of all species and census intervals that are surrounded at distance *r* by conspecific annular tree basal area load *b*. Finally, we estimate the difference


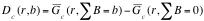
 (2)

between the mean growth rate of small trees without conspecific trees in annulus *r* (i.e., the sum of the basal areas *B* = 0) and that of small trees with conspecific trees in annulus *r* that sum up to total basal area *B* within *b*. This allows us to make a precise assessment of the relative effects of conspecific annular tree summed basal area load *b* at distance *r* on growth. If the difference *D*_C_(*r*, *b*) is negative, larger conspecific annular trees have, as expected for example from Janzen-Connell effects [[1](#_ENREF_1), [2](#_ENREF_2)], a negative neighborhood effect on the growth of the smaller focal trees.

Global averages are used in equations (1) and (2) because all the growth rates have been normalized within census periods. These equations are also employed with the subdivided data, in which species are grouped into subdivisions that have different abundances or different distributions of tree sizes. In these analyses, both focal and annular trees are confined to those belonging to the set of species being examined.

Equations (1) and (2) can be modified to yield the difference *D_P_* (*r*, *b*) between the growth rates of small focal trees in the presence or absence of trees of the summed basal area interval *b* in annulus *r* belonging to the group of species that lie within the specified phylogenetic distance range *P* from the species of the focal tree, *i.e.*, *G_P_ (r,b)*. This analysis explores how the growth rate of small focal trees is affected by the presence of larger annular trees that occupy different ranges of phylogenetic distances.

In the second class of growth rate analyses, which also use equations (1) and (2), we reverse the roles of small and large trees. These analyses address the question of whether the growth rates of large focal trees are affected by the presence of smaller annular trees. As noted above, in these analyses categories of annular trees are chosen that have summed basal areas smaller then the basal area of the focal tree.

In all these growth rate analyses, standard errors for the data from each annulus are estimated by performing unpaired t-tests on the two sets of growth rate data that are compared in Equation (2).

**(2) Analysis of annular tree aggregation.** In these analyses we total the mean numbers of conspecific annular trees and heterospecific annular trees in each of the annuli around focal trees of different sizes. This enables us to quantify how the neighborhood densities of conspecific or heterospecific neighbors around focal trees of size class *d* change with distance *r*. In each of the forest plots, only data from the second census period are used in the clustering analyses.

We denote the total number of focal trees of species *j* and size *d* as *F*_j_(*d*), and the total number of conspecific trees that were present at the start and end of the second census period in the annulus *r* around the focal trees as *S_C,j_*(*r*, *d*). Then the mean density of all conspecifics at distance *r* of focal trees in size class *d* is:


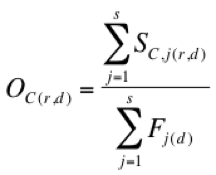
 (3)

Equation (3) yields the neighborhood density of trees in the annulus *r* around a focal tree of size *d* for this single census interval. As in the previous analyses, heterospecific survivors are obtained by substituting *S_P,j_*(*r*, *d*) for *S_C,j_*(*r*, *d*) in the equation, where *S_P,j_*(*r*, *d*) is the number of survivors of species that lie within a given phylogenetic distance interval *P* from the focal tree species.

These average numbers are compared using z-tests iof the differences with the distribution of average numbers obtained after 100 randomizations of focal tree sizes within species. This null model conserves the observed spatial structure of the tree placement, but removes any correlation with the sizes of the focal trees. It also ensures that any autocorrelations that arise from repeated use of the same annular trees are factored out because they are present in both the real data and the null model.

As with the focal tree growth analyses, total data and subdivided data were analyzed using these methods for each plot.

**(3) Analyses of annular tree recruitment.** In the EAA recruitment analyses we measure the mean fraction
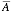
of conspecific or heterospecific recruits, relative to surviving trees, that are found in each annulus surrounding focal trees of a given size class, *i.e.*,
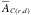
 or
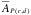
. Recruits are defined as trees that had at the beginning of the census interval a size below 1cm dbh and at the end of the interval a size larger than 1 cm. Survivors are defined as trees that had a dbh larger than 1 cm at both censuses.

In these analyses focal trees are divided into four size classes, 10-20, 20-50, 50-100 and >100 mm diameter, chosen to ensure that changes in recruitment that take place early in the life histories of the focal trees will be detected. Each recruitment analysis is carried out for all census periods, on the assumption that recruits in each census period are independent events.

In the conspecific recruitment analyses we first determine all focal trees of species *j* and size class *d* in each census interval *i*. This total includes focal trees that have no recruits and/or survivors within annulus *r*. We then estimate the total number *R*_C_*_,i,j_*(*r, d*) of conspecific recruits, and the total number *S*_C,_*_i,_*_j_(*r, d*) of conspecific survivors, in the annulus *r* around all the focal trees. Thus, the mean relative proportion of conspecific recruits to survivors among all trees within annulus *r* around focal trees of size class *d* (taken over all census intervals *i* and species *j*) yields


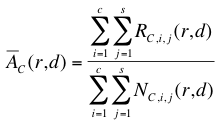
 (4)

Because the focal trees of all species are being summed, the numerator and denominator of this ratio are always greater than one.

This equation can be modified to measure phylogenetic influence on recruitment. We obtain the mean ratio of recruits to survivors among annular trees of species that lie within the range of phylogenetic distances *P* from the species of the focal tree. Again, these ratios are obtained for each annulus *r* around focal trees of size class *d* (taken over all census intervals *i* and species *j*).

Error bars are obtained by a null model that repeatedly shuffles the sizes of focal trees within each focal tree species, calculates the ratios of equation (4) after shuffling, and compares the mean of 100 sets of shuffled ratios with the actual ratio by a z-test.

**(4) Analyses of annular tree mortality.** In these analyses we measure differences in the mean fraction of conspecific or heterospecific mortality in the annuli that surround focal trees of different size classes. In the conspecific analyses we estimate the mean mortality rate
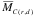
of conspecific annular trees at distance *r* of focal trees of size class *d*. This is the ratio of two numbers: in the numerator the total number *M_C,i,j_*(*r*, *d*) of conspecific trees in the annulus *r* around the focal trees of each size class *d* that died during the census period, and in the denominator the sum of this number and the number *S_C,i,_*_j_(*r*, *d*) of surviving conspecifics during the census period:


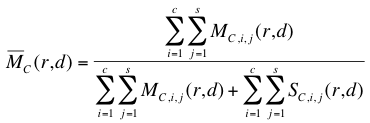
 (5)

Unlike the recruitment ratio, which is the ratio of recruits to survivors and which excludes trees that died during the census period, the mortality ratio is the fraction of the trees that died out of the total number of trees that were present in the annulus at the start of the census. For heterospecific analyses of phylogenetic effects the numbers of conspecific annular trees are replaced in equation (5) by the numbers of annular trees that belong to all species that lie within a specified phylogenetic distance interval *P* from the focal tree species. As in the clustering and recruitment data, the actual mortality ratios are compared by z-tests to 100 ratios in which focal tree sizes are shuffled within focal tree species.

**Text B. Details of the subdivision of the FDP data sets**

In the subdivision analyses our goal is to compare three groups of species with different properties, each group having equal amounts of data and therefore an equal likelihood of detecting the details of weak relationships between focal and annular trees. This enables us to ask whether or not the strength of the relationships, and their details, are comparable among the subsets.

In the species abundance subdivision, the plot data are divided into three equally-sized groups, made up of the most abundant species, those with intermediate abundance, and a large group of less abundant species. The first of the abundance subsets consists of the four most abundant species in each forest plot (*Hybanthus prunifolius, Faramea occidentalis, Trichilia tuberculata* and *Desmopsis panamensis* at BCI, and *Humboldtia laurifolia*, *Agrostistachys intramarginalis*, *Mesua nagassarium* and *Aporosa sp.* at Sinharaja). The next subset at BCI consists of the twenty-one next most abundant species, and at Sinharaja the sixteen next most abundant species. The rarest-species subset is made up of the remaining 295 (BCI) or 209 (Sinharaja) species.

In the tree-size-distribution subdivision, species are subdivided according to the coefficient of variation (CV) of their tree diameters, in order to distinguish between species that can achieve large sizes and those that remain small during their lifetimes. Again, our goal is to compare statistically equivalent subsets. The low-CV third consists of the 85 species with an average tree-size CV of 1.198 (BCI) and the 100 species with an average CV of 1.42 (Sinharaja). For the intermediate third, the equivalent values are 104 and 1.67 (BCI) and 76 and 1.40 (Sinharaja), and for the high third the values are 96 and 1.85 (BCI) and 49 and 1.77 (Sinharaja). As noted in the Methods section, the distributions of species among the abundance and tree-size-distribution subsets are largely independent of each other.


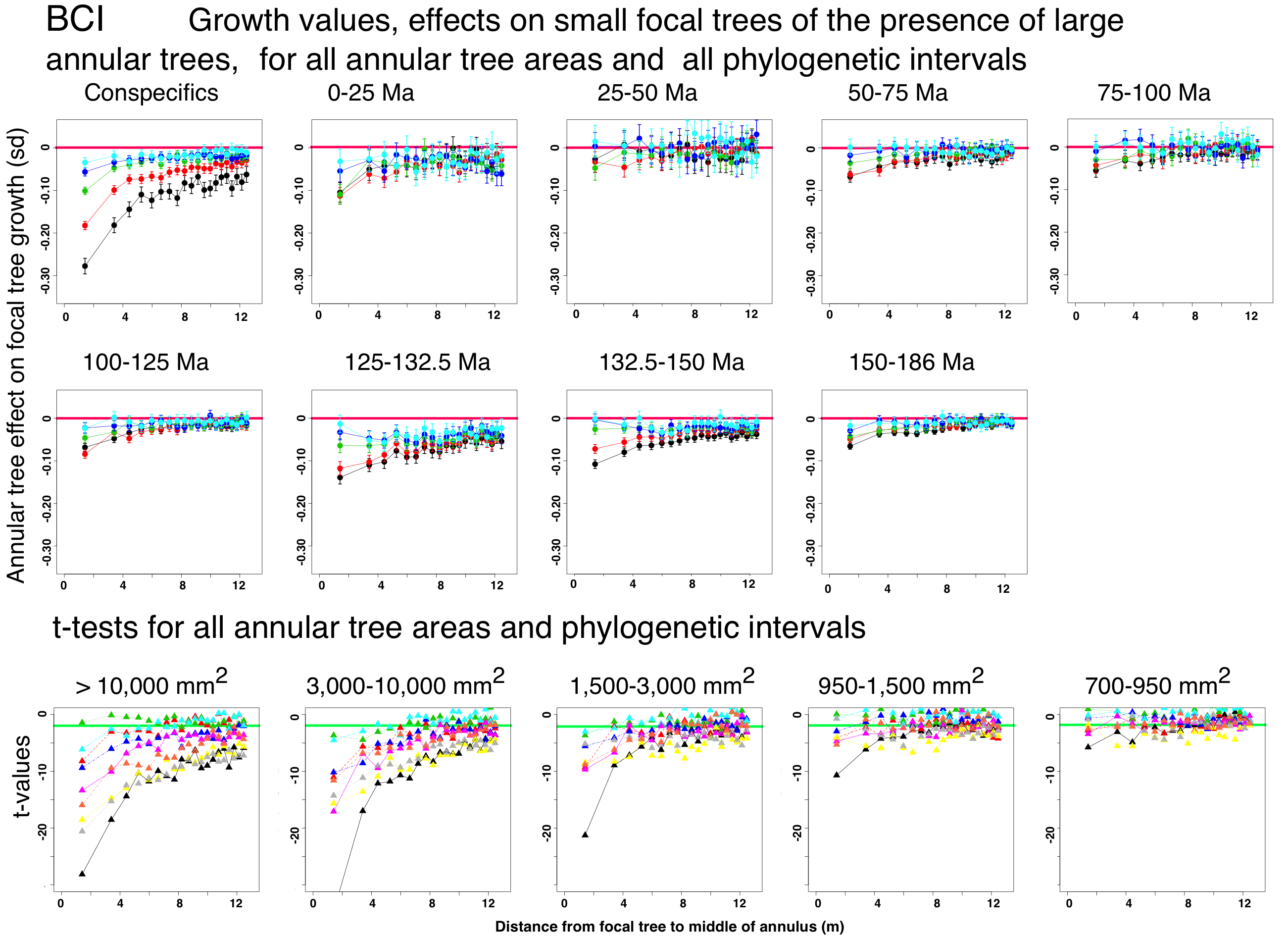


**Fig. A.** **BCI: Growth values for smaller focal trees in the presence of larger annular trees, for all annular tree basal areas and all phylogenetic distances.** The t-values are shown for the effects of the presence of all five size classes of larger annular trees. Legends as in Figs. 2 and 5.


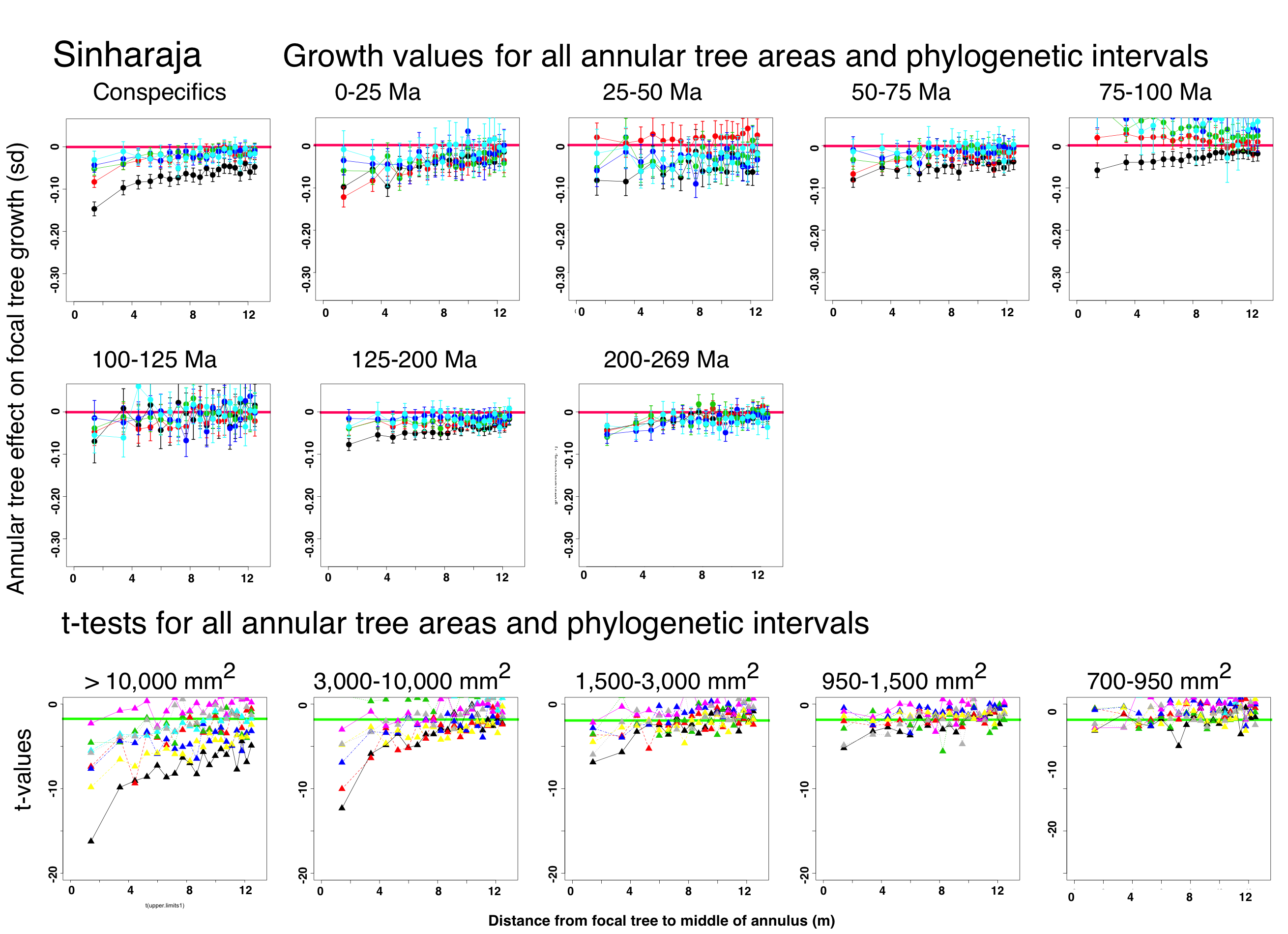


**Fig. B.** **Sinharaja: Growth values for smaller focal trees in the presence of larger annular trees, for all annular tree basal areas and all phylogenetic distances.** The t-values are shown for the effects of the presence of all five size classes of larger annular trees. Legends as in Figs. 2 and 5.


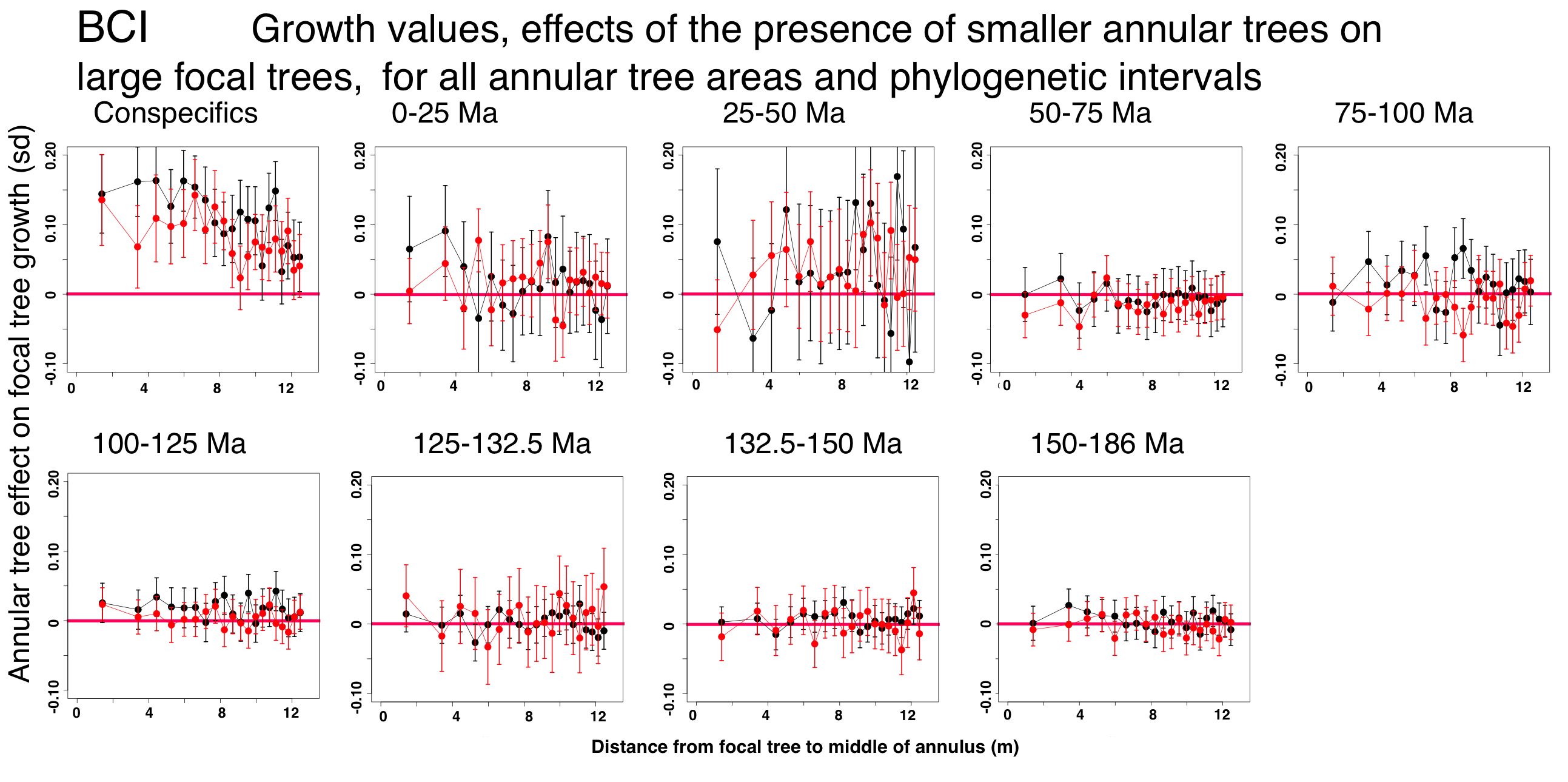


**Fig. C. BCI: Growth values for larger focal trees in the presence of smaller annular trees, for all annular tree basal areas and all phylogenetic distances.** Shown are the effects of two classes of annular trees with summed areas at base height that are smaller than the area at base height of the focal tree, for all phylogenetic intervals. Legend as in Figs. 6 and 9.


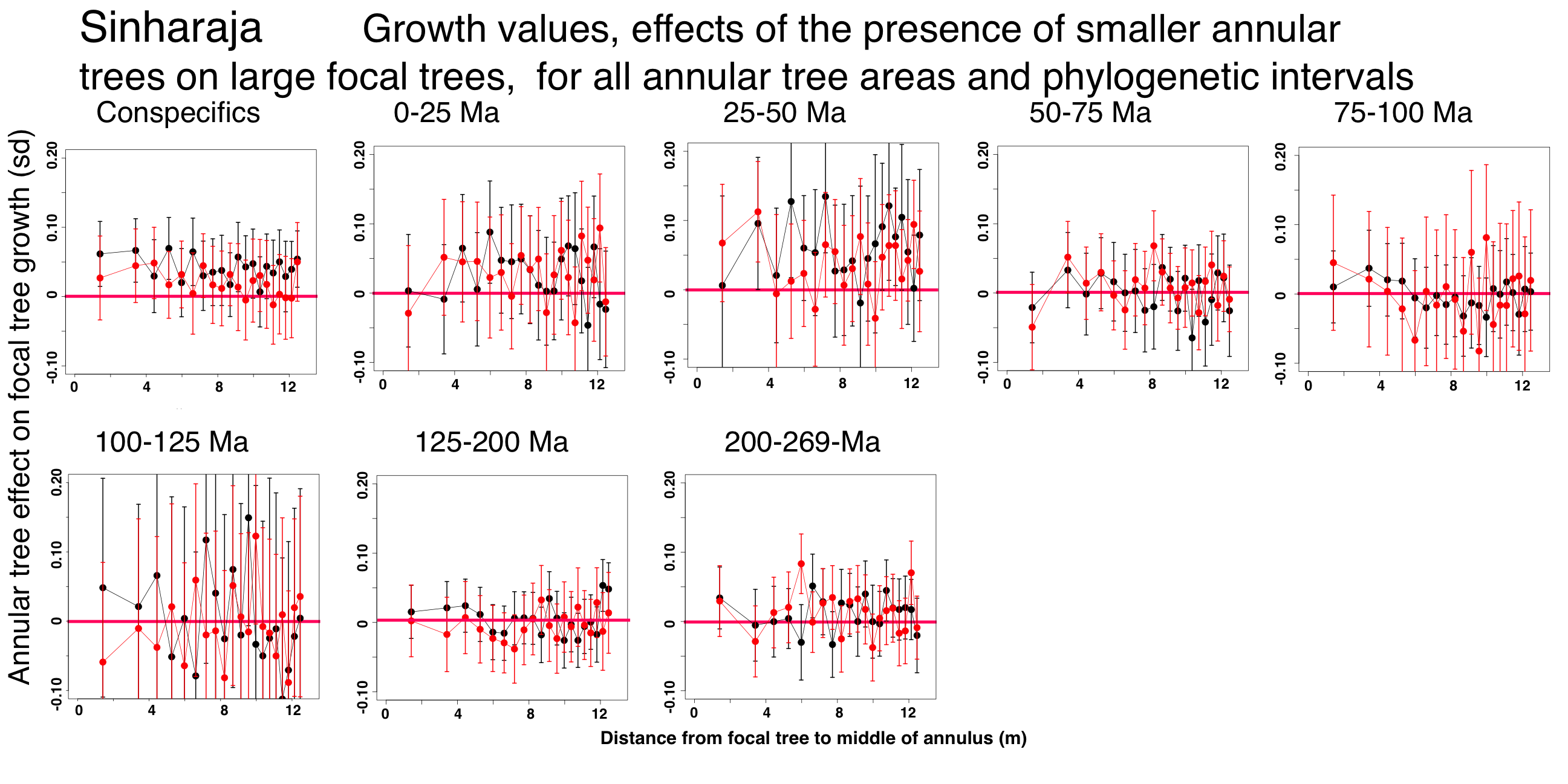


**Fig. D. Sinharaja: Growth values for larger focal trees in the presence of smaller annular trees, for all annular tree basal areas and all phylogenetic distances.** Shown are the effects of two classes of annular trees with summed areas at base height that are smaller than the area at base height of the focal tree, for all phylogenetic intervals. Legend as in Figs. 6 and 9.


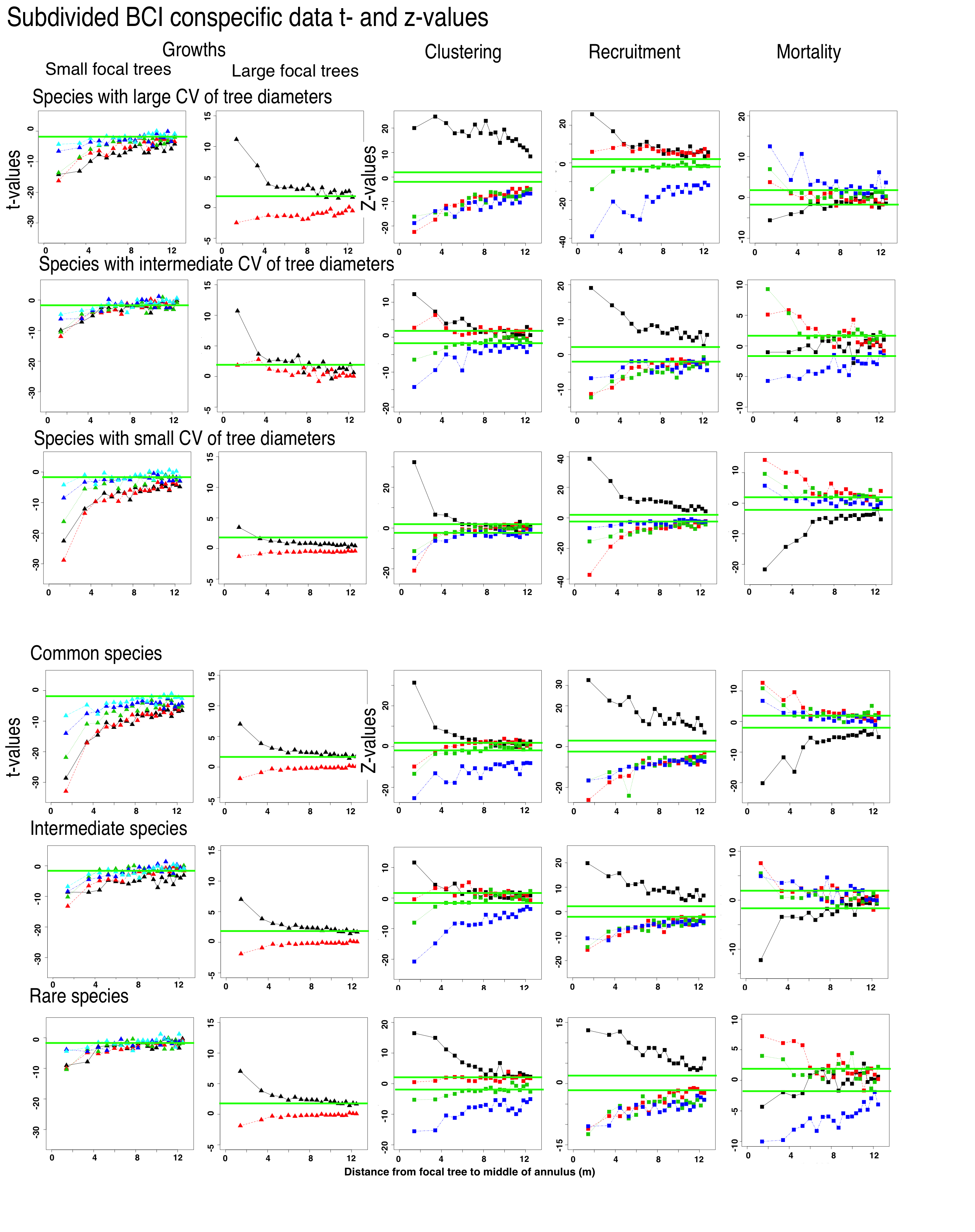


**Fig. E. BCI: Significances, presented as t-values and z-values, of the associations between focal trees of different size classes and their growth rates, and between their sizes and the degree of clustering, recruitment and mortality of their annular conspecifics, in all six subdivisions of the conspecific BCI data.** Horizontal green lines show the 95% confidence levels. Legends and axis labels as in Figs. 2 and 4.


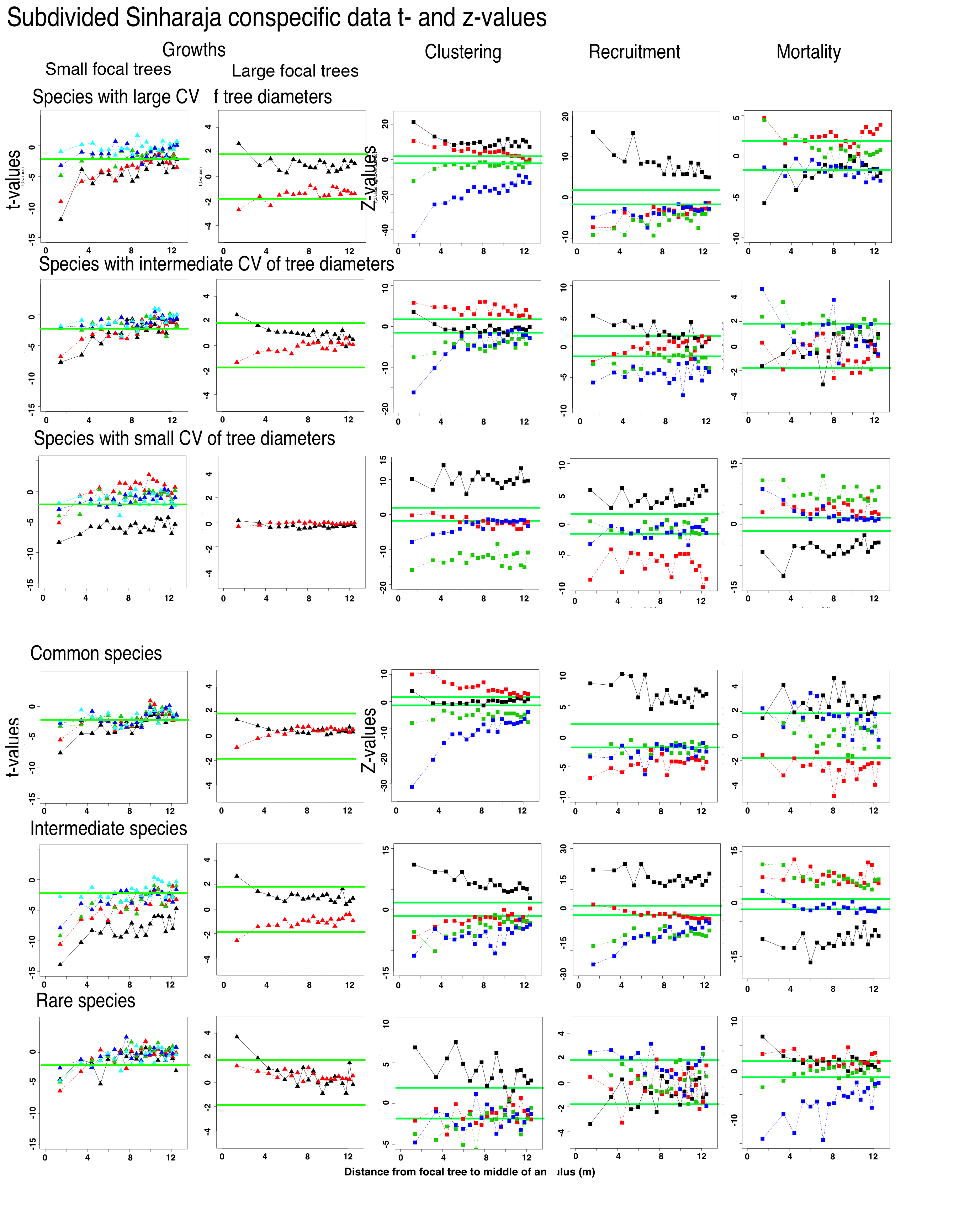


**Fig. F. Sinharaja: Significances, presented as t-values and z-values, of the associations between focal trees of different size classes and their growth rates, and between their sizes and the degree of clustering, recruitment and mortality of their annular conspecifics, in all six subdivisions of the conspecific Sinharaja data.**

Horizontal green lines show the 95% confidence levels. Legends and axis labels as in Figs. 2 and 4.

**References for supplemental material**

1. Janzen DH. Herbivores and the number of tree species in tropical forests. Am Nat. 1970;104:501-29.

2. Connell JH. On the role of natural enemies in preventing competitive exclusion in some marine animals and in rain forest trees. In: Den Boer PJ, Gradwell G, editors. Dynamics of Populations. New York: PUDOC; 1971. p. 298-312.
