# Supplementary material for: Correction: Persistence of Neighborhood Demographic Influences over Long Phylogenetic Distances May Help Drive Post-Speciation Adaptation in Tropical Forests
Source: PLoS One. 2016 Dec 20;11(12):e0168976. doi: 10.1371/journal.pone.0168976 (PMC5173372; doi:10.1371/journal.pone.0168976)
Supplement: S1 File — (PDF) [file pone.0168976.s001.pdf]

PLOS | ONE

RESEARCH ARTICLE

Persistence of Neighborhood Demographic Influences over Long Phylogenetic Distances May Help Drive Post-Speciation Adaptation in Tropical Forests

Christopher Wills[1]*, Kyle E. Harms[2], Thorsten Wiegand[3,4], Ruwan Punchi-Manage[5], Gregory S. Gilbert[6,7], David Erickson[7], W. John Kress[7], Stephen P. Hubbell[7,8], C. V. Savitri Gunatilleke[9], I. A. U. Nimal Gunatilleke[9]

1 Section of Ecology, Behavior and Evolution, Division of Biological Sciences, University of California San Diego, La Jolla, California 92093–0116, United States of America, **2** Dept. of Biological Sciences, Louisiana State University, Baton Rouge, Louisiana 70803, United States of America, **3** Dept. of Ecological Modeling, UFZ-Helmholtz Centre for Environmental Research GmbH–UFZ, 04318 Leipzig, Germany, **4** German Centre for Integrative Biodiversity Research (iDiv) Halle-Jena-Leipzig, Biodiversity Synthesis, Deutscher Platz 5e, 04103 Leipzig, Germany, **5** Department of Ecosystem Modelling, University of Göttingen, Büsgenweg 4, 37077 Göttingen, Germany, **6** Dept. of Environmental Studies, University of California Santa Cruz, Santa Cruz, California 95064, United States of America, **7** Smithsonian Tropical Research Institute, Balboa, Ancón, Panamá 0843–03092, **8** Dept. of Ecology and Evolutionary Biology, University of California Los Angeles, Los Angeles 90095, California, United States of America, **9** Dept. of Botany, Faculty of Science, University of Peradeniya, Peradeniya, 20400 Sri Lanka

* cwills@ucsd.edu

CrossMark
click for updates

OPEN ACCESS

Citation: Wills C, Harms KE, Wiegand T, Punchi-Manage R, Gilbert GS, Erickson D, et al. (2016) Persistence of Neighborhood Demographic Influences over Long Phylogenetic Distances May Help Drive Post-Speciation Adaptation in Tropical Forests. PLoS ONE 11(6): e0156913. doi:10.1371/journal.pone.0156913

Editor: RunGuo Zang, Chinese Academy of Forestry, CHINA

Received: February 23, 2016

Accepted: May 20, 2016

Published: June 15, 2016

Copyright: This is an open access article, free of all copyright, and may be freely reproduced, distributed, transmitted, modified, built upon, or otherwise used by anyone for any lawful purpose. The work is made available under the Creative Commons CC0 public domain dedication.

Data Availability Statement: BCI plot data are available from http://ctfs.arnarb.harvard.edu/webatlas/datasets/bci/. The Sinharaja Forest Monitoring Plot data belongs to the plot PIs and is managed by the CTFS-ForestGEO network. Data are available under request at http://ctfs.si.edu/Public/plotdataaccess/SiteDescription.php?plotname=sinharaja&typedata=tree.

Funding: Thorsten Wiegand and Ruwan Punchi-Manage acknowledge funding from ERC advanced grant 233066-SPATIODIVERSITY. The funders had

Abstract

Studies of forest dynamics plots (FDPs) have revealed a variety of negative density-dependent (NDD) demographic interactions, especially among conspecific trees. These interactions can affect growth rate, recruitment and mortality, and they play a central role in the maintenance of species diversity in these complex ecosystems. Here we use an equal area annulus (EAA) point-pattern method to comprehensively analyze data from two tropical FDPs, Barro Colorado Island in Panama and Sinharaja in Sri Lanka. We show that these NDD interactions also influence the continued evolutionary diversification of even distantly related tree species in these FDPs. We examine the details of a wide range of these interactions between individual trees and the trees that surround them. All these interactions, and their cumulative effects, are strongest among conspecific focal and surrounding tree species in both FDPs. They diminish in magnitude with increasing phylogenetic distance between heterospecific focal and surrounding trees, but do not disappear or change the pattern of their dependence on size, density, frequency or physical distance even among the most distantly related trees. The phylogenetic persistence of all these effects provides evidence that interactions between tree species that share an ecosystem may continue to promote adaptive divergence even after the species' gene pools have become separated. Adaptive divergence among taxa would operate in stark contrast to an alternative possibility that has previously been suggested, that distantly related species with dispersal-limited distributions and confronted with unpredictable neighbors will tend to converge on common

no role in study design, data collection and analysis, decision to publish, or preparation of the manuscript.

Competing Interests: The authors have declared that no competing interests exist.

strategies of resource use. In addition, we have also uncovered a positive density-dependent effect: growth rates of large trees are boosted in the presence of a smaller basal area of surrounding trees. We also show that many of the NDD interactions switch sign rapidly as focal trees grow in size, and that their cumulative effect can strongly influence the distributions and species composition of the trees that surround the focal trees during the focal trees' lifetimes.

Introduction

The many forest dynamics plots (FDPs) that are being maintained in primary forests around the world, under guidance from the Center for Tropical Forest Science (CTFS) of the Smithsonian Tropical Research Institute and with the cooperation of governmental and research institutions, have allowed ecologists to follow the histories and fates of millions of individual trees [1, 2]. Repeated censuses of some of the FDPs at 5-year intervals have provided detailed information about changes over time in these species-rich ecosystems.

There is growing evidence for the importance of density-dependent effects, particularly negative density-dependence (NDD), in the maintenance of species diversity in forests [3] and [4], and in other complex ecosystems such as tropical reefs [5]. These effects tend to be strongest within individual species—species tend to be at a competitive advantage when locally rare and lose that advantage when they are locally common. In this paper we examine these effects in detail in two tropical forest plots and show that they extend even to the most distantly related species that share these ecosystems. Although the strength of the effects drops rapidly with increasing phylogenetic distance between tree species, we will show that they have the capability of forcing continued evolutionary change. Even distantly related tree species will continue to diverge from each other in the direction of sharing fewer physical resources and fewer parasites and pathogens, provided that the tree species share an ecosystem.

One explanation for NDD patterns is the Janzen-Connell model, which proposes that species-specific pathogens and seed-predators accumulate around trees as they mature and reduce the survival of the trees' seeds and seedlings [6–9]. In a number of important studies, distance- and density-dependent effects on conspecific seedling survival have been traced to the effects of pathogens (e.g. [10–12]). If trees' growth rates are positively correlated with their survival and ability to reproduce, negative density-dependent effects of surrounding trees on growth that may also be traceable to the activities of pathogens should also contribute to the maintenance of species diversity. Uriarte et al. [13, 14] found conspecific NDD effects of immediate neighborhoods on trees' growth rates in the BCI FDP.

An alternative but not mutually exclusive explanation for conspecific NDD effects is niche-complementarity, in which the density- and distance-dependent effects result from the species-specific depletion of physical resources [15–17]. A third model is facilitation, in which benefits, mediated through a wide variety of biotic mechanisms, accrue to organisms of a given species from the presence of nearby heterospecific organisms [18].

Although many of these NDD effects have been examined in previous studies of a wide variety of FDPs and grasslands (e.g. [8, 12, 19–32]), most of the studies have involved conspecific plants. The extent and patterns of heterospecific interactions have been less thoroughly studied.

It is known that distantly-related tree species share fewer pathogens than closely-related ones, e.g. [33–35]. Zhu and co-workers [36] found a pattern consistent with decreased

pathogen sharing with phylogenetic distance: a weaker NDD signal for recruitment in heterospecific than in conspecific trees in a survey of 50 temperate tree species in the eastern United States. Seedlings surrounded by closely-related trees are less likely to die than those surrounded by distantly-related trees [3, 37, 38].

Recently, Zhu et al. [38] examined survival of the 29 commonest tree species in the BCI FDP. They found that survival of small trees is reduced, and that of larger trees is increased, in the presence of conspecific neighbors. They then took the average of the phylogenetic distances between a tree and all of its surrounding heterospecific neighbors within a series of radii—5, 10, 15 or 20 m. If a tree's heterospecific neighboring trees are on average closely related to it, their effect on its survival is similar to, but weaker than, that of conspecific neighbors. This latter effect is only apparent in trees larger than saplings. Chen et al. [39] have found a small positive effect of phylogenetic distance of surrounding trees on the absolute non-standardized growth rates of focal trees, though this effect was smaller than the effect of physical dissimilarities between species.

In this paper we explore details of the phylogenetic persistence of a wide range of negative density-dependent and distance-dependent (NDD) effects among saplings and reproductive trees in the BCI and Sinharaja plots. These include (1) associations between a tree's growth rate and size and the species composition and total basal area of its neighbors, and (2) associations between a tree's size and the amounts of recruitment, mortality and clustering in its neighborhood. To the extent that these effects are present in heterospecific tree species interactions and are negatively density-dependent, they will force the tree species to become more different from each other.

Although other studies have shown that focal seedling growth, recruitment and mortality are affected by the species composition and distribution of their surrounding trees [4, 13, 22, 34, 40, 41], and seedlings have been shown to be more strongly affected than larger trees [42], our study is the first to extend these observations to all size categories of focal trees. We show in detail the extent to which these effects are strongly dependent on tree size, show that the effects begin to change early in a tree's life history, and demonstrate that they sometimes change sign from negative to positive density dependence as trees mature. We are the first to show that in both FDPs the characteristics of all of the effects examined, including their tree size dependence, persist even when they are measured between individual trees and surrounding trees that are separated from them by hundreds of millions of years of divergent evolution.

The present study examines a wider variety of NDD effects over a wider range of phylogenetic distances than previous studies (see Methods). We find that the effects diminish rapidly with increasing phylogenetic distance, but that the characteristics of the effects, such as the strong dependence on focal tree size, persist even in larger trees and when there are extremely large phylogenetic separations between species. Because this consistent pattern of persistent phylogenetic signals in many NDD effects is found in both the BCI and Sinharaja FDP plots, we predict that it may be detectable in other forests.

Data and Methods

Data sets

The BCI plot on Barro Colorado Island in Panama and the Sinharaja plot in Sri Lanka, 50 and 25 ha respectively, are located in tropical wet forests. The available census data exclude lianas and "strangler" figs but include all other woody plants $\geq$ 10 mm diameter at breast height (dbh) [43]. The BCI plot (9°10′N, 79°51′W) receives 2,600 mm rain per year, has a pronounced 3.5-month dry season from January to April, and has a year-round mean daily temperature of 27°C [44]. Topographic relief ranges from 120 to 155 m. The Sinharaja plot (06° 24′ N, 80° 24′ E) receives

an annual average of 5,000 mm of rain and has a less pronounced dry season lasting from December to April [45]. Topographic relief is more pronounced at Sinharaja, with elevations ranging from 424 to 575 m above mean sea level. Annual daily maximum and minimum temperatures are 24.7° and 20.4°C, respectively. The BCI plot has been censused seven times, beginning in 1982, and the Sinharaja plot has been censused three times, beginning in 1996. Except for the first census interval at BCI, which spanned the years 1982–85, the censuses in the plots have been carried out at 5-year intervals.

Analysis of phylogenetic distances

To assess the impact of evolutionary relationships among species on NDD traits, a community phylogeny of the tree species in the BCI plot was generated using a multi-locus DNA barcode library of three chloroplast markers (*rbcL*, *matK*, and *trnH-psbA*). Details of sequence editing, alignment, and assembly are provided in Kress *et al.* [46]. The resulting phylogeny was then dated with the program PATHd8, following Magallon and Castillo [47] for dates at nodes of specific orders to convert the phylogeny into a dated chronogram. Evolutionary distances between pairs of taxa in the Sinharaja plot were estimated using the Magallon and Castillo divergence times of 265 genera in 52 orders and families, based on sequences of three plastid genes (*atpB*, *rbcL*, and *matK*) and two nuclear markers (18S nuclear ribosomal DNA [nrDNA] and 26S nrDNA). Branch lengths were adjusted with the *Phylocom* statistical package [48].

Equal-area annulus (EAA) method

The EAA neighborhood density function that we introduce here is an extension of O-ring spatial statistics [49] and the Dx index [50]. It is a non-parametric point-pattern analysis that examines associations between the species, size, and normalized growth rate of individual "focal" trees and a range of properties of the "annular" trees that fall within a series of twenty equal-area (25 m^2) concentric annuli surrounding each focal tree. Unlike parametric approaches such as maximum likelihood neighborhood analyses [51], it is not model-dependent. The equal-area annulus approach also ensures that comparable amounts of data are available for analysis in each annular subdivision. Details of the EAA analysis are presented in Text A of S1 File.

EAA and other spatial point pattern analyses also differ from quadrat-based analyses (e.g. [19, 21]) in that they can examine properties of the surroundings of individual trees rather than the averaged properties of small focal areas of the plots. Their summary statistics typically use information from the same tree multiple times [52, 53]. While this can lead to problems with autocorrelation in regression analysis, this is circumvented through the use of non-parametric Monte Carlo null models to test for significance. Autocorrelations are equally present in the observed and the null model data, and factor out when they are compared. Details of the Monte Carlo methods used are presented in Text A of S1 File.

Here we employ the EAA method to examine focal tree growth rate in the presence or absence of conspecific and heterospecific annular trees. We also analyze how the degree of clustering, recruitment, and mortality of annular conspecific and heterospecific trees changes—and often changes sign—as focal trees increase in size. These latter analyses show in detail how the environment of focal trees undergoes rapid changes early in their lifespans. We also show that these effects, like the effects on focal tree growth rate, diminish but do not disappear with increasing phylogenetic distance between focal and annular tree species.

We designate as focal trees all trees that survive a given census period, that are at least 12.6 m from the edge of the plots, and that belong to species in which there are at least 20 trees present in the entire plot during each census interval. Fig 1 shows the pattern of the twenty

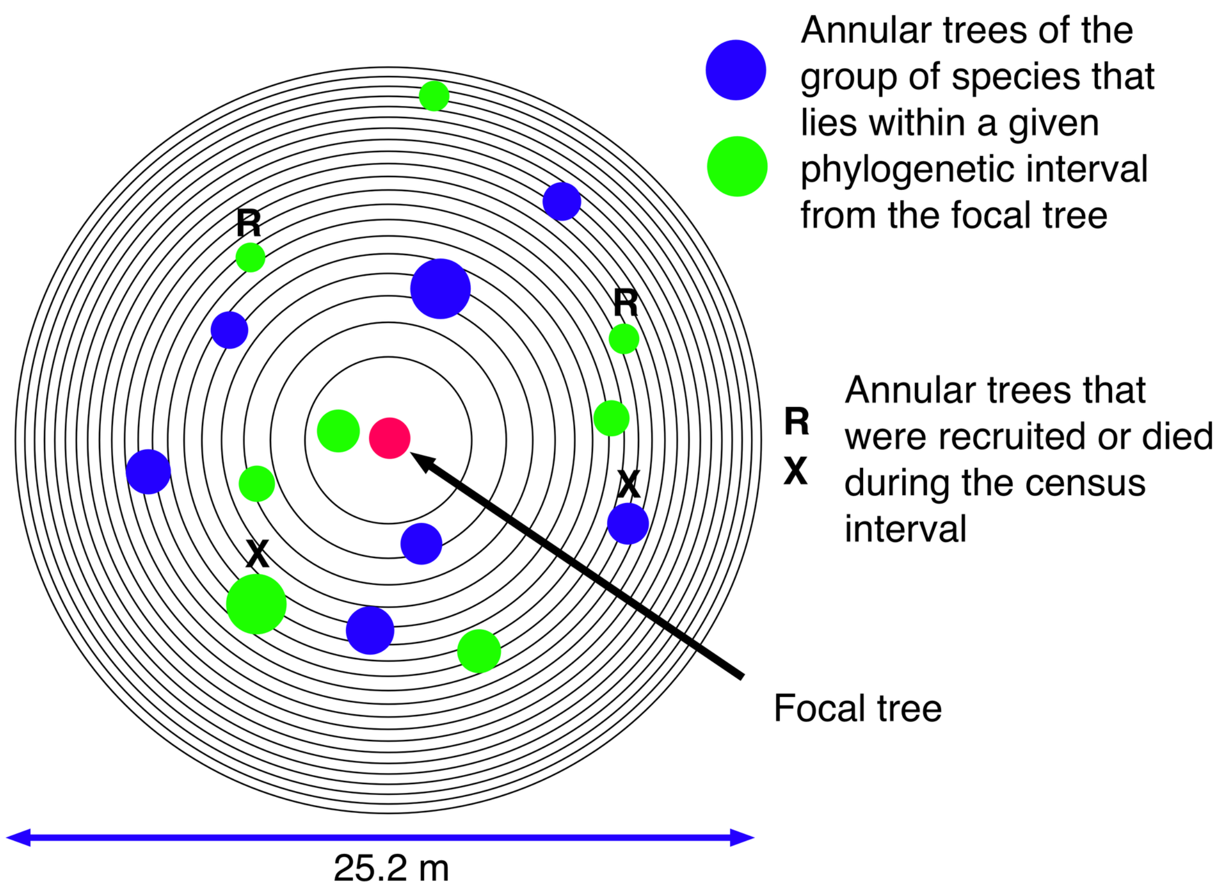

Fig 1. A map of the 20 equal-area concentric annuli around each focal tree that were used in the EAA analysis. The figure represents the annuli around a focal tree during a single census period, and shows annular trees of two species (green and blue symbols) that fall within a specified phylogenetic interval from the focal tree (red symbol). R and X designate annular trees that were recruited or died during the census period.

doi:10.1371/journal.pone.0156913.g001

equal-area annuli surrounding each focal tree. The outer margin of the first annulus is 2.8 m from the focal tree, and the inner and outer margins of the 20th annulus are 12.3 and 12.6 m from the focal tree. Thus, the focal trees are all far enough from the edges of the plot that all annuli surrounding them are complete, eliminating the need for edge corrections. The BCI and Sinharaja plot data are analyzed separately.

Focal trees are characterized by their species, dbh, and normalized growth rate, calculated by dividing the trees into ten dbh classes delimited by diameters of 10, 30, 50, 70, 90, 110, 130, 150, 170, 190, and >190 mm. Each tree's normalized growth rate is expressed as the number of positive or negative standard deviations by which the tree's growth rate deviates from the mean growth rate of its size class within its species.

The trees that occupy each annulus are divided into conspecifics of the focal tree and heterospecifics. The heterospecifics are in turn divided into groups in which the annular trees fall within the following ranges of phylogenetic distances (in Ma) from their common ancestor with the focal tree: 0–25, 25–50, 50–75, 75–100, 100–125, 125–132.5, 132.5–150 and 150–186 for BCI and 0–25, 25–50, 50–75, 75–100, 100–125, 125–200 and 200–269 for Sinharaja. These numerous intervals ensure that detailed conclusions can be drawn about the effects of evolutionary divergence. All of the subdivisions are made up of more than 750 focal-annular pairs of tree species, providing a substantial number of pairwise comparisons between the focal tree

and its related trees in each annulus. Each of these phylogenetic groupings of heterospecifics in each annulus are examined to determine their numbers, rates of recruitment and survival, and summed basal areas at breast height.

We have examined the growth rate of small focal trees (dbh 10–30mm) in the presence or absence of five categories of larger conspecific (or phylogenetically related) trees in each of its annuli. We conducted similar analyses for growth rate of large focal trees (dbh > 190 mm) in the presence or absence of two categories of smaller annular trees. We have also examined conspecific (or phylogenetically related heterospecific) clustering, recruitment and mortality in all the annuli around focal trees of different size classes (10–20, 20–50, 50–100 and >100 mm dbh).

Growth rates, recruitment, and mortality in succeeding census periods are assumed to have a substantial independent component, so that data from all census periods are used. In the clustering analysis, because annular tree numbers and species composition for the same focal tree are strongly correlated across censuses, only data from the second census periods of each of the plots are used.

Clustering is estimated as the density of annular trees, either conspecifics or trees falling within a given phylogenetic interval from the focal tree, that are present at both the beginning and end of the census period. The recruits in an annulus during a census period are all individuals of conspecifics, or of species that lie within a given phylogenetic interval from the focal tree, that cross the size threshold of 1 cm during that census period. The recruitment rate in the annulus is estimated as the mean fraction of recruits among conspecific survivors or survivors that fall within the given phylogenetic interval. The mortality rate is the fraction of conspecifics, or trees falling within the phylogenetic interval, that are present at the start of the census period and that die during that period.

We have pooled information from all of the tree species in the BCI or in the Sinharaja plots in our initial analyses, in order to maximize the likelihood of detecting even weakly significant relationships between the properties of focal and annular trees. Following this initial analysis, we have carried out preliminary tests of whether or not subgroups of species with different properties show the same pattern of relationships as the plot as a whole. In these tests the plot data are partitioned into large subsets, each containing equal numbers of focal trees. Analysis of each subset therefore has equal statistical power. Each subset is made up of species that share a particular property: either similar abundance or similar distribution of tree sizes. Other groupings, such as canopy and understory species, could be made, but they would not permit the division of the data into equal fractions. Each subset includes both the focal and the annular trees, so that the EAA analyses of a subset employ only the focal-annular relationships between the species that make up the subset. Details of the subdivision process are given in Text B of S1 File.

Measuring the significance of NDD effects and the degree to which they are independent of the effects of topography and within-quadrat autocorrelation. The NDD effects examined here, even the relatively strong effects between conspecific focal and annular trees, explain only a small fraction of the total variances of focal tree properties. Further, these variances are influenced by other factors.

One of these factors is plot topography. A second possible confounding factor is the presence of autocorrelations. Trees that are near each other will, for a wide variety of reasons, tend to have similar growth, recruitment and mortality rates.

To quantify the topographic effects, we employ classifications of the topographies of 20 x 20 m quadrats of the plots. These topographic classifications have been shown in previous studies (BCI, [54], Sinharaja, [55] to be strongly related to species assemblages and to shifts in assemblage composition as trees mature. BCI plot quadrats are divided into four different categories:

slope, low plateau, high plateau or swamp. Sinharaja quadrats are divided into five categories (high elevation, mid elevation, low elevation with high altitude above channel, and low elevation with low altitude above channel). We give each quadrat a designation that corresponds to one of the topographic categories, and assign each focal tree in a quadrat the designation of that quadrat.

To aid in quantifying autocorrelations between the properties of trees located physically near each other we assign different numbers to the 20 x 20 m quadrats, and assign all the focal trees in the quadrat that number. We have examined four response variables, along with sets of factors that would be expected to influence each variable, and the interactions between the factors. The first of these variables and factor sets is: (1) growth rates of small focal trees vs. three factors: presence or absence of larger annular conspecific trees in the first annulus, quadrat topographic designation, and quadrat number. The other three response variables are: (2) number of conspecific surviving trees during the second census period in the first annulus, (3) number of conspecific recruits in the first annulus around focal trees during each census period, and (4) number of conspecific trees that die during each census period in the first annulus. We estimate the response of each of these variables to the same set of three factors: focal tree diameter, quadrat topography, and quadrat number.

Results

EAA Analysis Results

The depression in small focal tree growth rates in the presence of annular conspecifics is strongly correlated with the basal area of the annular conspecifics. Our conspecific growth rate analysis examines the effect of the presence or absence of certain categories of conspecific annular trees on the growth rate of small (10–30mm dbh) focal trees. For each of the 20 annuli, we divide the focal trees into five groups on the basis of the summed basal areas of the conspecifics that are present in the annulus. We use the growth rate of small focal trees that do not have conspecific neighbors in the annulus as a point of reference. We then average, across all species of focal tree, the differences between the normalized growth rates of focal trees with summed basal area *b* of conspecifics (observed effect) and basal area 0 (point of reference) in a given annulus.

In both forest plots the presence of a large summed basal area of annular trees is associated with a highly significant slowing of growth of small focal trees (Fig 2). The greatest effects are seen for the growths of small focal trees that have the largest summed basal area of conspecifics in an annulus (black color in the figure). The size of these effects diminishes with decreasing size difference between focal and annular trees and with increasing distance of the annuli from the focal tree. These patterns are also observed for other focal tree sizes (data not shown). In general the growth rate of focal trees of any size class is slowed in the presence of a larger summed basal area of annular conspecifics.

Growth effects diminish with increasing phylogenetic distance between focal and annular trees. Fig 3 shows the equivalent measurements of small focal tree growth in the presence or absence of closely-related heterospecific annular trees, again across all the categories of summed basal area of annular trees and across all 20 annuli. Fig 4 shows the effect of the presence or absence of the most distantly related annular heterospecifics. In both forest plots there is a negative effect of the presence of a larger basal area of annular heterospecifics on small focal tree growth, and the effect is more pronounced in the closely-related species. Unpaired t-tests (Fig 5) between the sets of focal tree growth rates in the presence or absence of annular trees show that the growth effect is significant in most of the nine (BCI) or eight (Sinharaja) categories of phylogenetic intervals between focal and annular trees. Although the levels of

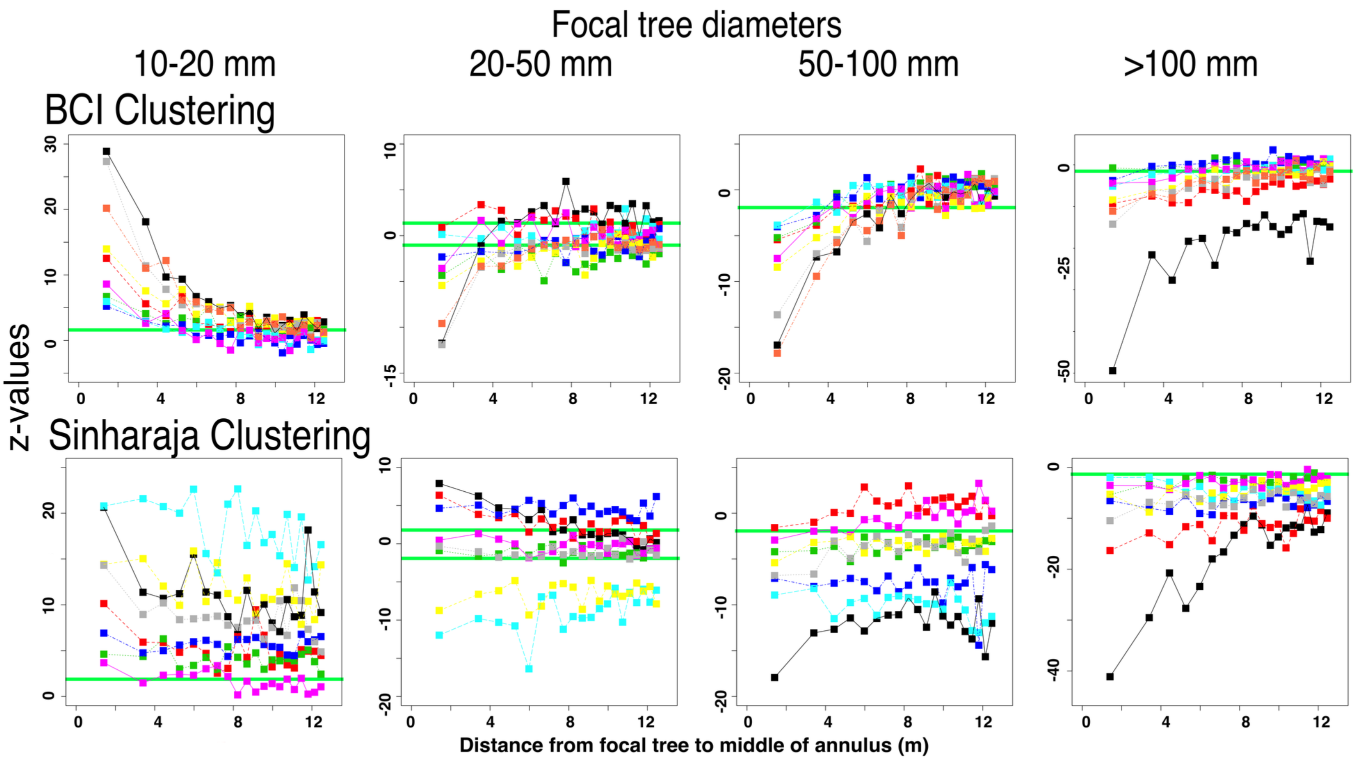

Fig 2. Negative effects on the growth rates of small (dbh 10–30 mm) focal trees that are associated with the presence of a range of sizes of conspecific trees in the annuli. For each of the 20 annuli, the normalized growth rates of small (diameter 10–30 mm) focal trees in the presence of larger conspecific annular trees are averaged. The figure shows the differences between these averages and the averaged growth rates of focal trees that have no conspecific trees in the annulus (in standard deviations). Panels A and B present data from BCI and Sinharaja, and the legend shows the range of sizes of summed annular tree basal areas that were used in the analysis. Horizontal red lines denote zero difference between the mean growth rates. Error bars are derived from unpaired t-tests of the sets of growth values.

doi:10.1371/journal.pone.0156913.g002

significance vary for the different phylogenetic intervals, in part as a function of the amount of information available, the growth effect remains significant even at the most distant phylogenetic interval.

Figs A and B of S1 File show, for the two plots, all the growth effects on focal trees associated with the presence or absence of heterospecific annular trees that fall into all five categories of summed basal areas and all the categories of phylogenetic intervals between focal and annular trees. The figures also summarize the results of unpaired t-tests between the distributions of focal tree growths in the presence or absence of the different categories of annular trees. These tests show that the largest growth rate effects are found when the annular trees are physically and phylogenetically close to the focal trees, and when the size differences between the focal and annular trees are large.

Growth rates of large focal trees increase in the presence of smaller annular trees. Fig 6 shows an unexpected result. In both forest plots, the mean growth rate of the largest focal trees (dbh > 190 mm) is increased if a slightly smaller summed basal area of annular conspecifics is nearby, compared with the mean growth rate of the largest trees in the absence of any annular conspecifics. This result may reflect processes that have led to the increased survival of large trees surrounded by conspecifics that was detected by [38]. This effect, like the effects shown in Figs 3 and 4, also falls off with phylogenetic distance (Figs 7 and 8), but it remains significant at BCI even at large phylogenetic distances (Fig 9).

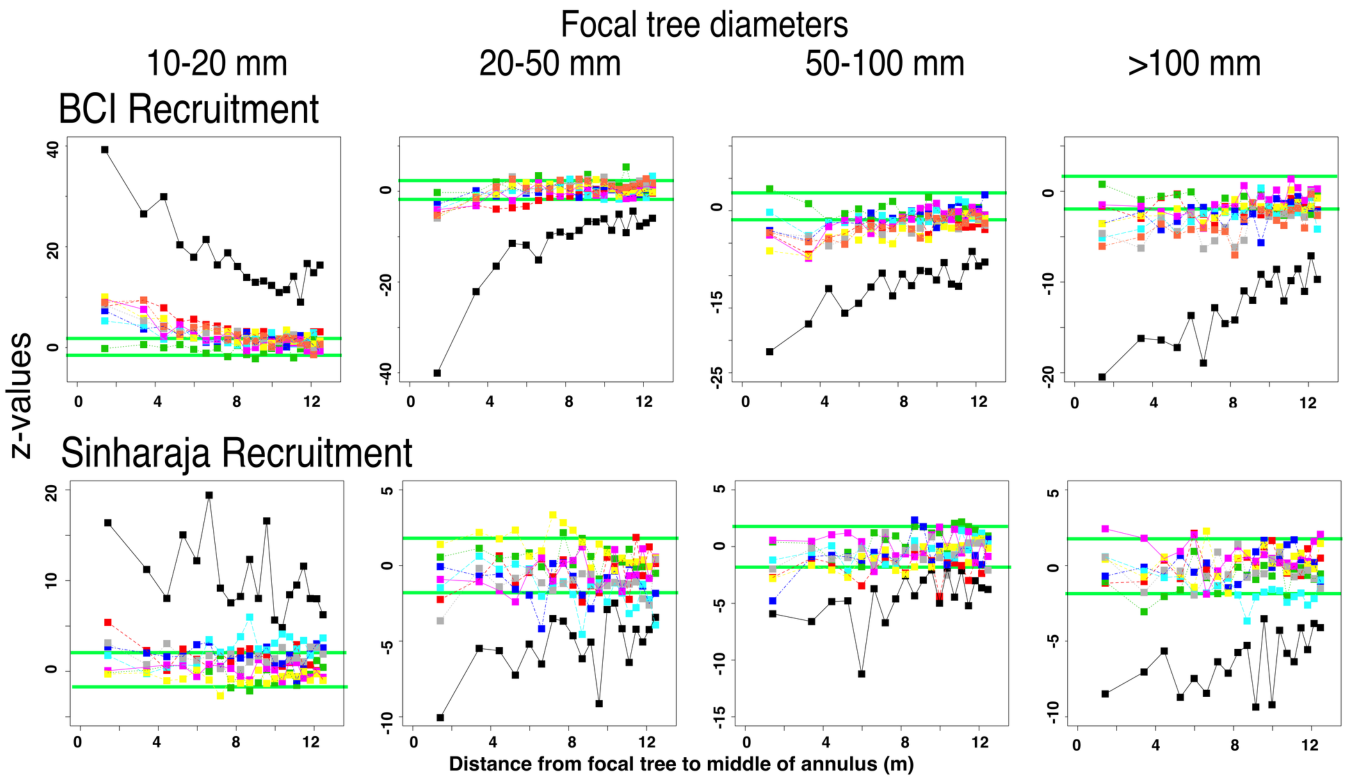

Fig 3. Effects on the growth rates of small (dbh 10–30 mm) focal trees in the presence and in the absence of annular heterospecific trees of species that are closely related to the focal tree. The annular trees consist of the species that lie within the interval 0–25 Ma since the time of their last common ancestor with the focal tree species. Data presented as in Fig 2.

doi:10.1371/journal.pone.0156913.g003

Figs C and D of S1 File show the complete data for large focal tree growths, for both plots and for all phylogenetic intervals. At BCI, the t-values for annuli that are close to the focal tree are significant at all phylogenetic intervals. At Sinharaja the level of significance at all phylogenetic intervals is substantially lower, but there are still some significant effects at large phylogenetic intervals.

Density dependence in clustering, recruitment and mortality. Many studies have shown that tree recruitment and mortality in FDPs is consistent with negative density-dependent effects on life history parameters. Our EAA analyses of clustering, recruitment and mortality examine these life history parameters from a different point of view. We ask how, and how quickly, the surroundings of an individual tree change as the tree grows in size. The answer is: surprisingly quickly. Small trees of 10–20 mm dbh are surrounded by a significantly higher than average density of conspecifics, and among these conspecifics recruitment is higher and mortality is lower than average. But by the time trees become slightly larger, 20–50 mm dbh, their surroundings have undergone a substantial change. Conspecifics have thinned out to a lower than average density, the rate of recruitment has dropped, and the rate of mortality has risen. These deviations from the mean often become more pronounced as focal trees grow further in size. We show further that all these patterns persist across even the largest phylogenetic distances between focal and annular trees (orange and gray symbols in the figures for BCI and Sinharaja respectively). In sum, the characteristics of the trees surrounding focal trees that survive and grow are clearly different from those of the trees surrounding the smallest focal trees.

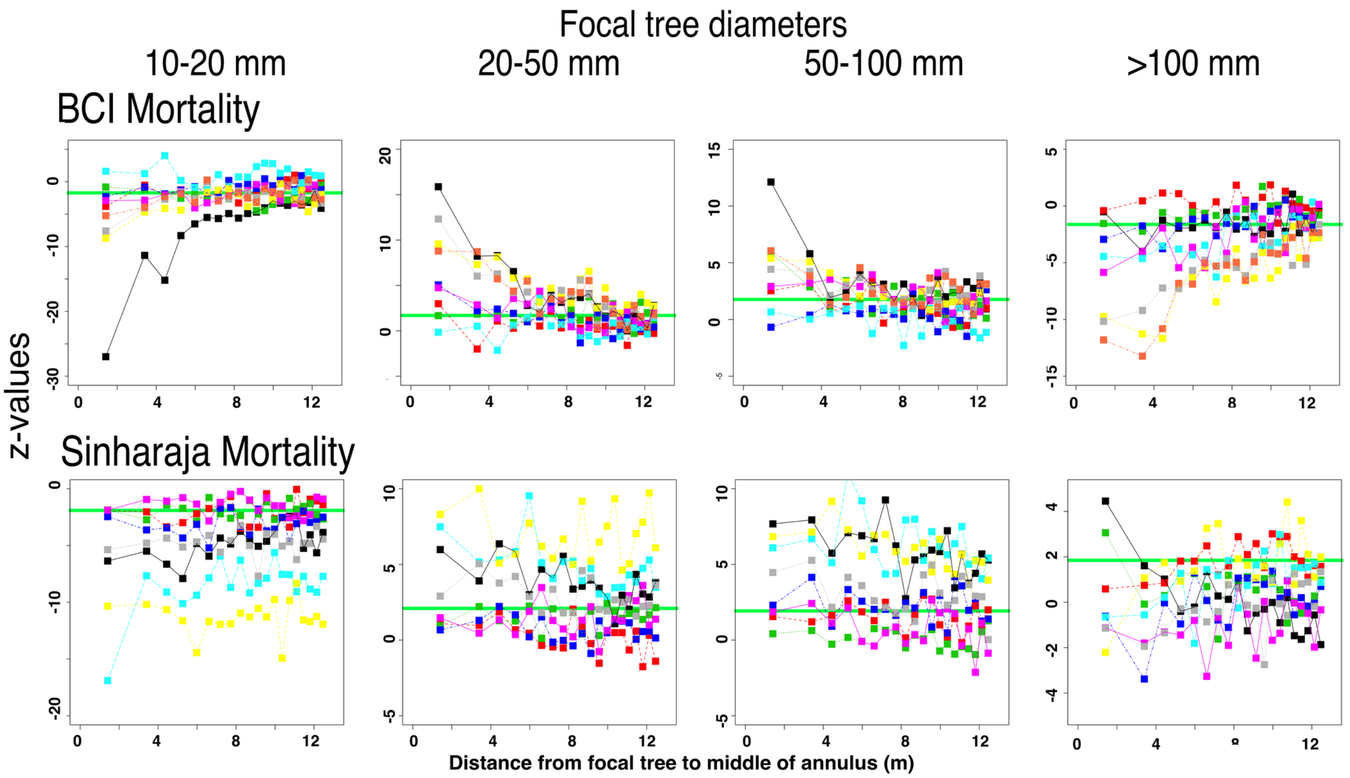

Fig 4. Differences between the normalized growth rates of small (dbh 10–30 mm) focal trees in the presence and in the absence of annular heterospecific trees that are distantly related to the focal tree. The annular trees consist of the species that lie within the interval 150–186 Ma (BCI) or 250–269 Ma (Sinharaja) since the time of their last common ancestor with the focal tree species. Data presented as in Fig 2.

doi:10.1371/journal.pone.0156913.g004

Clustering of annular conspecifics is greatest around small focal trees, and quickly changes to overdispersal with increasing focal tree size. An important finding to emerge from the analyses of species-rich forest dynamics data was the discovery that tree species tend to be aggregated rather than overdispersed [56]. Rarer species also tend to be more aggregated than common ones, and aggregation is more pronounced at Sinharaja than at BCI [50]. In our EAA analyses, the degree of aggregation is strongly associated with focal tree size. Fig 10 shows the significance of these values, compared with the null model in which focal tree sizes have been repeatedly (100 x) randomized within species. A positive z-value indicates that the observed clustering in the annulus is higher than expected from the null model. A negative z-value indicates that the annular trees are overdispersed. The figure shows that conspecific annular trees exhibit strong aggregation near focal trees of 10–20 mm dbh, low aggregation around focal trees of 20–50 mm dbh, and strong overdispersal around focal trees that are larger than 50 mm dbh.

The figure also shows that in both FDPs these patterns extend beyond conspecifics and can be detected at most phylogenetic distances between focal and annular species. As noted earlier, aggregation was measured only for the second census interval at each plot.

Recruitment rates of annular conspecifics are highest around small focal trees, and decrease swiftly with increasing focal tree size. Fig 11 shows z-values for recruitment rates of annular trees in the two FDPs, compared with the null model in which focal tree sizes have been repeatedly randomized within species. Recruitment tends to be depressed near large focal

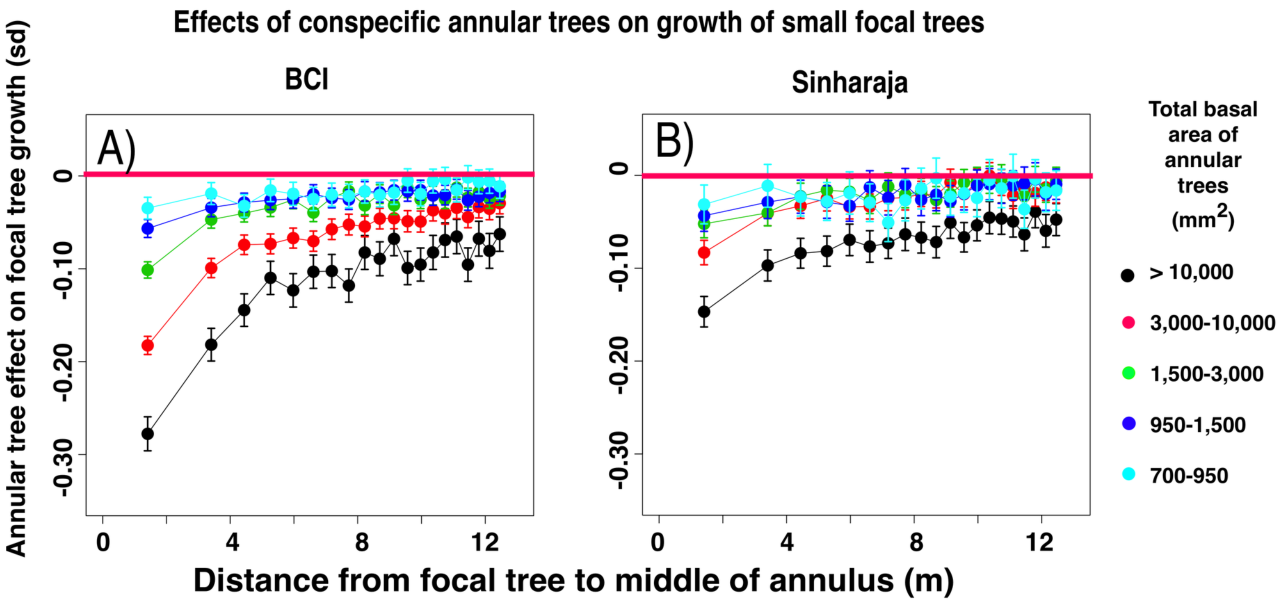

Fig 5. Significance of differences between the normalized growth rates of small (diameter 10–30 mm) focal trees in the presence and in the absence of larger annular heterospecific trees that are related to the focal tree, for all phylogenetic intervals between focal and annular tree species. The significances are measured as unpaired t-tests of the difference between the sets of growth rates of focal trees in the presence or absence of the designated subclass of annular trees. Horizontal green lines represent 95% significance levels. The legend shows the ranges of phylogenetic intervals between focal and annular tree species that were examined in each of the FDPs.

doi:10.1371/journal.pone.0156913.g005

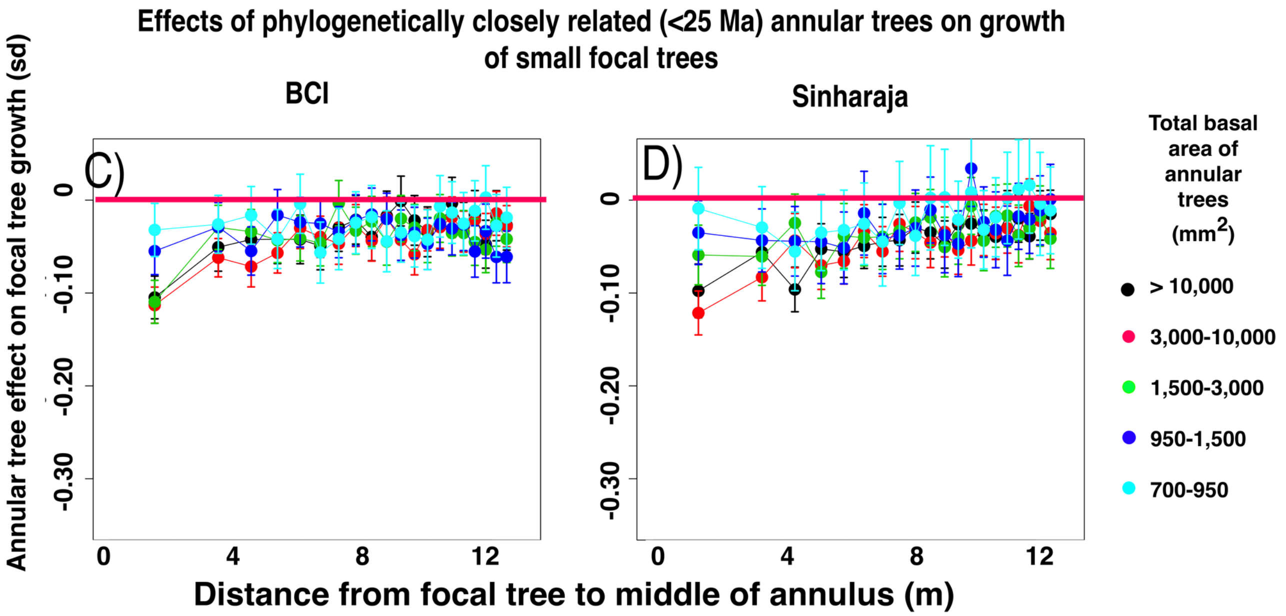

Fig 6. Positive effects on the growth rates of large focal trees (> 28,000 mm^2 dbh) that are associated with the presence of a range of sizes of conspecific trees in the annuli. As in Fig 2, panels A and B present data from BCI and Sinharaja, and the legend shows the range of sizes of summed annular tree basal areas that were used in the analysis.

doi:10.1371/journal.pone.0156913.g006

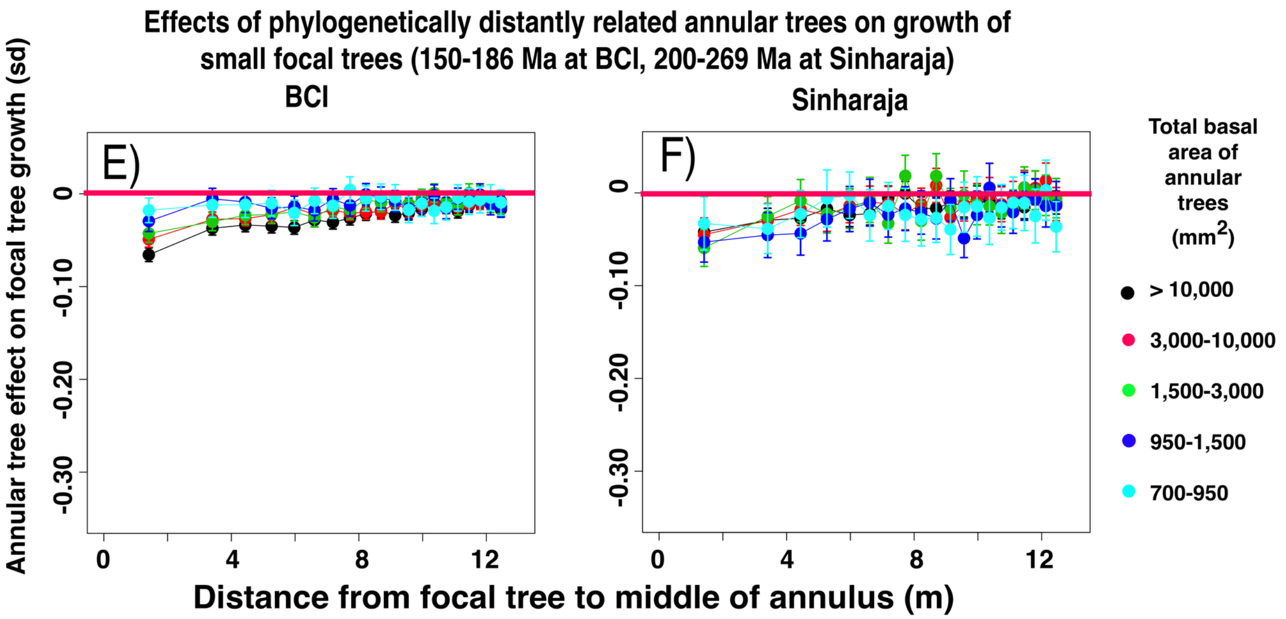

Fig 7. Effects on the growth rates of large focal trees (> 28,000 mm^2 dbh) in the presence and in the absence of annular heterospecific trees of species that are closely related to the focal tree. The annular trees consist of the species that lie within the interval 0–25 Ma since the time of their last common ancestor with the focal tree species. Data presented as in Fig 2.

doi:10.1371/journal.pone.0156913.g007

trees, because of a variety of factors ranging from Janzen-Connell effects to the depletion of species-specific physical and biotic resources [40, 42, 57]. We find that a switch from excess to lowered recruitment of surrounding trees becomes apparent early in the life cycle of the focal trees. The observed fraction of recruits near focal trees >20 mm dbh is less than null-model

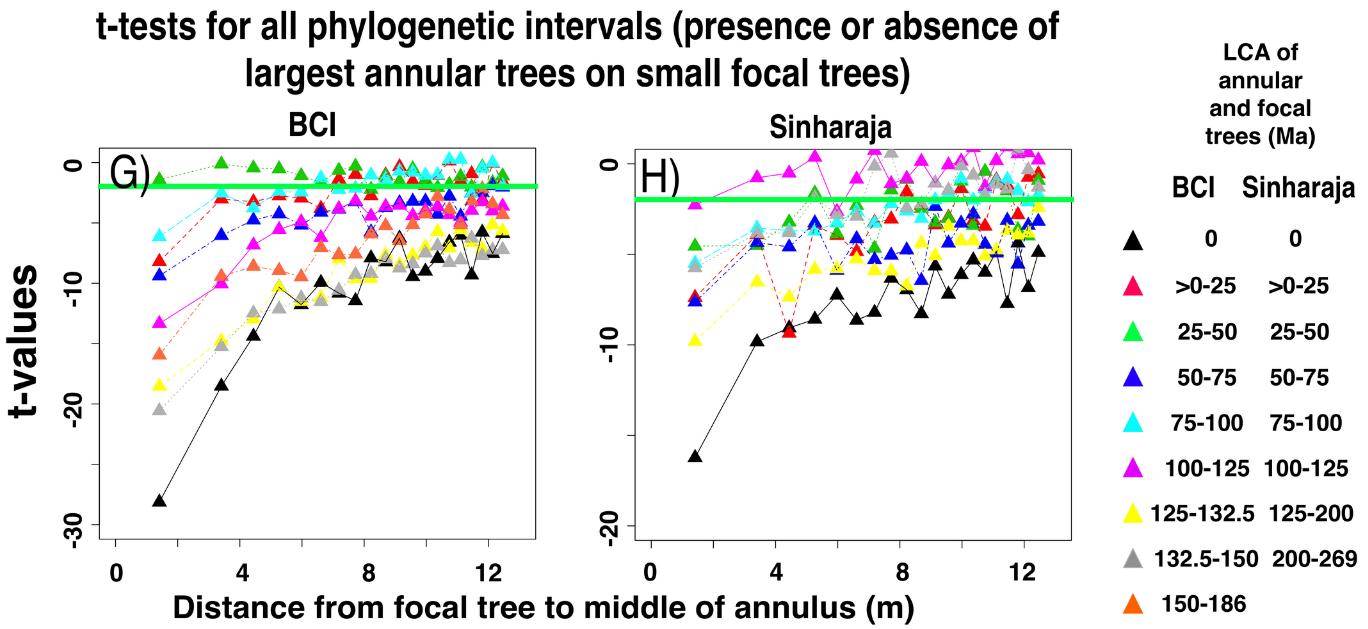

Fig 8. Differences between the normalized growth rates of large focal trees (> 28,000 mm^2 dbh) in the presence and in the absence of annular heterospecific trees that are distantly related to the focal tree. The annular trees consist of the species that lie within the interval 150–186 Ma (BCI) or 250–269 Ma (Sinharaja) since the time of their last common ancestor with the focal tree species. Data presented as in Fig 2.

doi:10.1371/journal.pone.0156913.g008

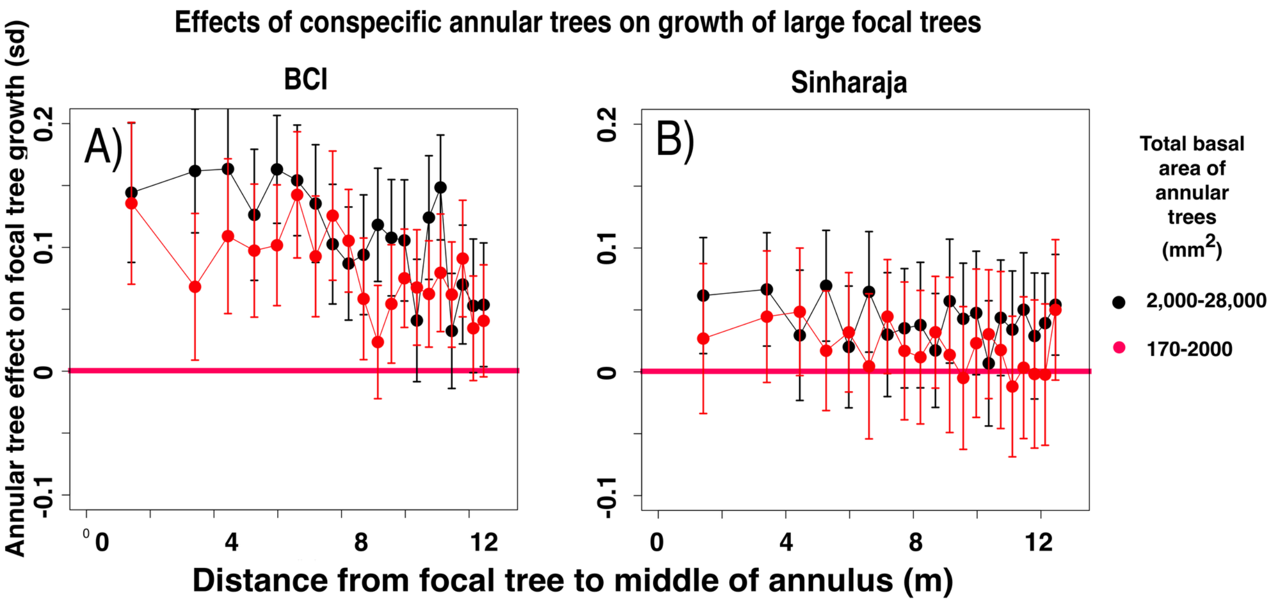

Fig 9. Significance of differences between the normalized growth rates of large focal trees (> 28,000 mm^2 dbh) in the presence and in the absence of annular conspecific trees or heterospecific trees that are related to the focal tree, for all phylogenetic intervals between focal and annular tree species. Data presented as in Fig 5.

doi:10.1371/journal.pone.0156913.g009

values in both forest plots. The figure also shows that similar decreases of annular recruitment with increasing focal tree size are apparent at most phylogenetic distances between focal and annular trees. The only exception to this pattern is an excess of recruits at large phylogenetic distances around the largest (>100 mm) category of focal trees at Sinharaja.

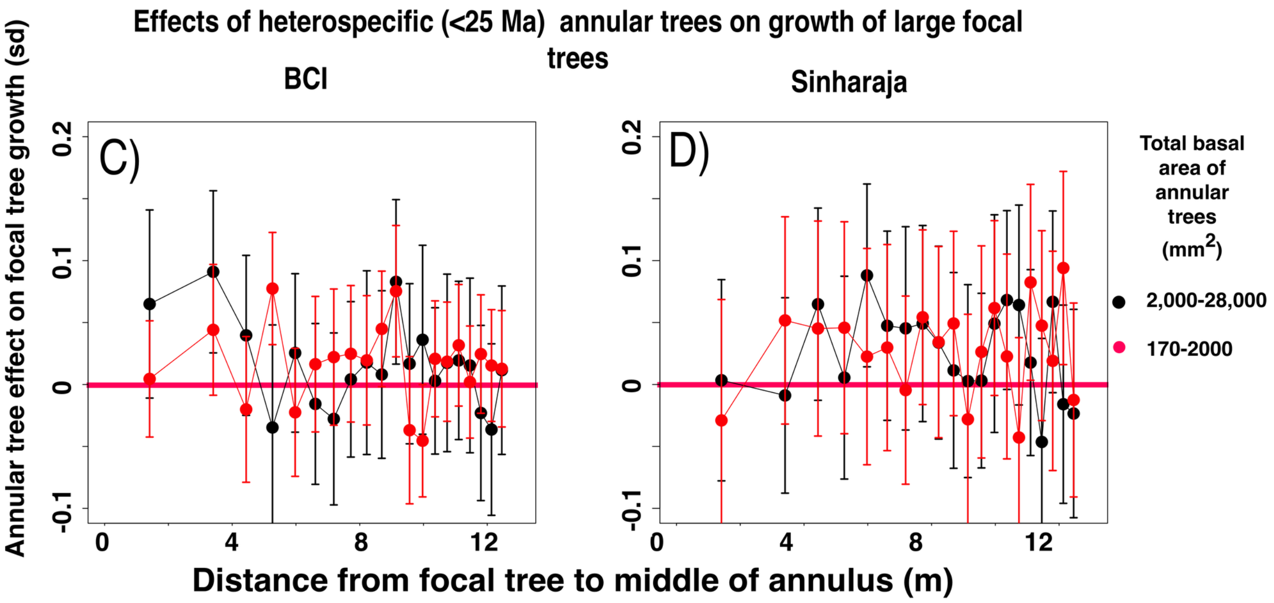

Fig 10. The significance of clustering of annular trees around focal trees of different sizes, showing how the pattern of significance changes with increasing focal tree dbh. Significance is measured as z-values of the differences between the real data mean and the means of 100 otherwise identical data sets in which the focal tree dbh values have been shuffled within species. All phylogenetic intervals between focal and annular trees are shown, denoted as in the legend to Fig 5.

doi:10.1371/journal.pone.0156913.g010

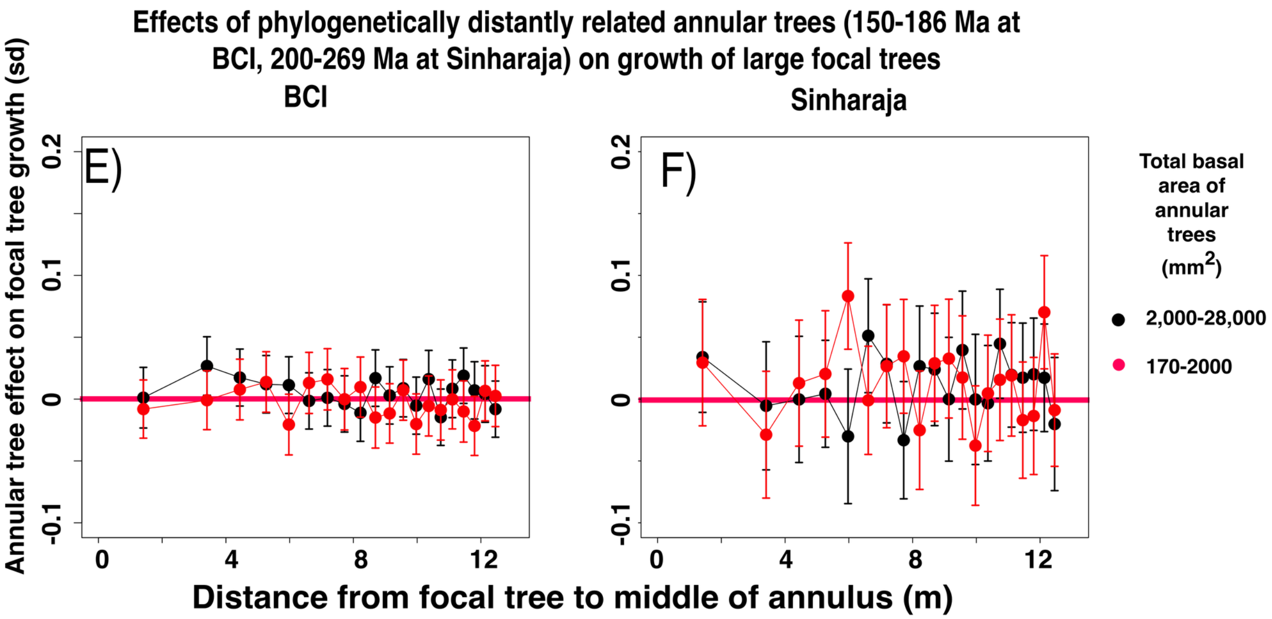

Fig 11. **The significance of proportions of recruits in annular trees around focal trees of different sizes, showing how the pattern of significance changes with increasing focal tree dbh.** Data presented as in Fig 10.

doi:10.1371/journal.pone.0156913.g011

The mortality rates of annular trees also show patterns of rapid change as small focal trees increase in size. Fig 12 shows z-values for annular mortality rates around focal trees of different sizes. In both forest plots, mortality exhibits a pattern that is the mirror image of recruitment. We find, as NDD models predict and in agreement with earlier observations of seedling mortality [3, 4], that mortality of trees larger than seedlings is significantly lower than

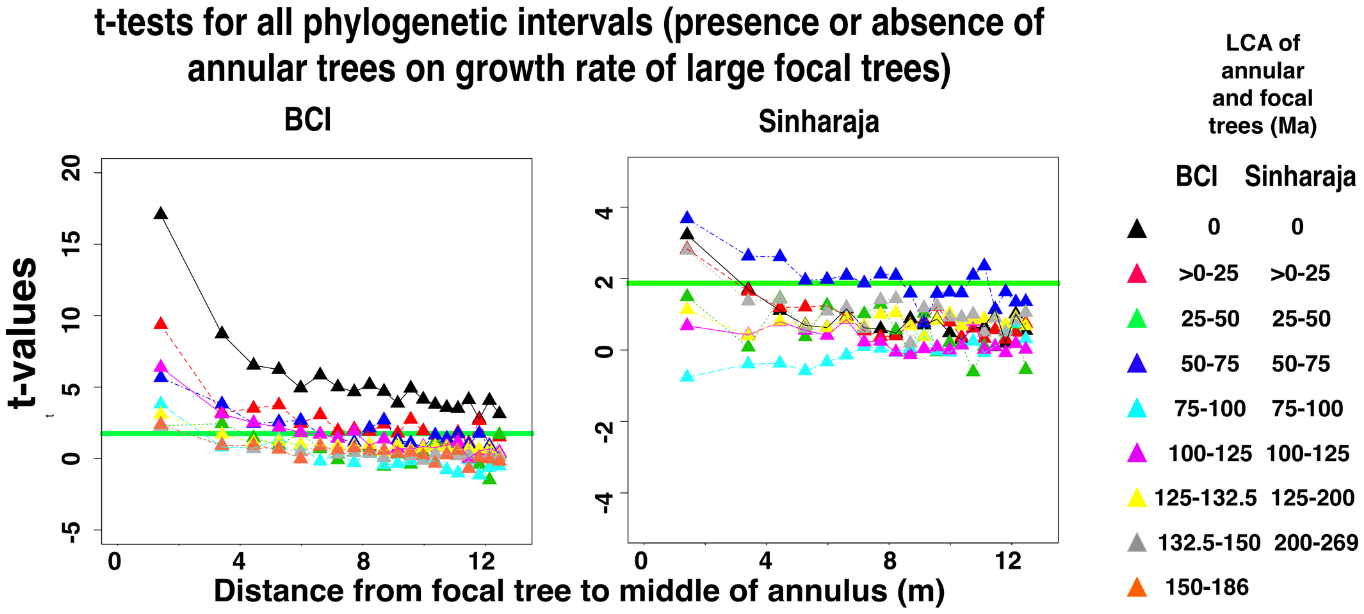

Fig 12. **The significance of mortality rate in annular trees around focal trees of different sizes, showing how the pattern of significance changes with increasing focal tree dbh.** Data presented as in Fig 10.

doi:10.1371/journal.pone.0156913.g012

expected near small focal trees. But we also find that mortality of surrounding trees is higher than expected near even slightly larger focal trees. As in the recruitment data, this switch from deficiency to excess mortality takes place between focal tree size categories of 10–20 mm and 20–50 mm diameter, indicating a rapid and early rise in surrounding mortality that starts early in the focal trees' life cycle.

Subdivisions of the data sets show relationships between focal and annular trees that resemble those of the full data sets. The effects presented above are based on pooled data for all the species in each of the FDP plots. Conclusions drawn from such multi-species pooling would not be justified if the species in the plots exhibit a wide diversity of properties. Further, most of the species are rare and cannot be analyzed to the same level of detail as the commonest species. We therefore asked whether two independent sets of analyses, based on subdivision of the data into thirds on the basis of different properties of the species, would yield results similar to the pooled analyses (Text B of S1 File).

The subdivisions were made on the basis of species abundance and on the basis of the CV of within-species tree size. On average, 31.6% of the species in an abundance subdivision were also found in a CV subdivision, with a minimum of zero and a maximum of 60.6%. All of the conspecific analyses that were performed on the entire data set were repeated using each of these species subgroups. Our goal was twofold: first, to ensure that all the analyses of the subsets have equal statistical power; and second, to test the hypothesis that the relationships found in the entire data set are also found in the subsets.

The results of the t-tests and z-tests on these subsets are shown in Fig E (BCI) and Fig F (Sinharaja) of S1 File. Without exception, growth differences in each of the subdivisions of the data resemble those that are found in the pooled data, though as expected the significance levels are lower. And, as with the pooled data, in almost all cases the smallest trees in each of the subdivided data sets have a significant excess of annular conspecifics and conspecific recruits, and a significant deficiency of conspecific mortality. The only exceptions are the intermediate CV and rare species mortality data at BCI, and the common species mortality data at Sinharaja, which show weakly significant effects that are only detectable in comparisons between the smallest focal trees and their surrounding conspecifics.

Analysis of Variance Results

Effects associated with plot topography and within-quadrat autocorrelations are largely independent of size-, distance- and density-related effects. Topographic subdivisions of the FDPs have been found to be strongly associated with characteristic tree species assemblages at the high-relief Sinharaja plot [55, 58]. Weaker but still significant associations between topography and species assemblages have been found at the low-relief BCI plot [59]. The extent of possible interactions between these topographic effects and size-, density- and distance-related effects was not, however, determined in those studies. We were concerned that such interactions, if they are substantial, might affect the size and significance of the relationships that we detect using the EAA analyses. A similar potential problem is presented by autocorrelations between the life history characteristics of pairs of focal trees that are in close physical proximity. These factors, which range from shared physical and biological characteristics of the local environment to possible sharing of genotypes between nearby trees, might also influence our results.

Tables 1–4 shows the results of analyses of variance carried out on four combinations of response variables and sets of factors for both forest plots (see Methods). The ANOVA data, in agreement with the t- and z-value data presented in Figs 2–6, show that the relationships between focal tree growth and the summed basal areas of larger annular conspecifics, as well as

Table 1. Analyses of variance that examine the relationships between the response variable of focal tree growth and the factors of annular tree basal area, focal tree size, plot topography, and focal tree autocorrelation. In this analysis, and in the analyses of Tables 2–4, the topographic factor is the topographic classification of the 20 x 20 m quadrat in which the focal tree is located, and the autocorrelation factor is the extent to which properties of focal trees are correlated when the focal trees share the same quadrat. Because of constraints in available computer programs that limit the number of interaction terms that can be estimated in an analysis, it was only possible to obtain interaction mean squares and F-values for 400 of the 1250 BCI quadrats and 400 of the 625 Sinharaja quadrats. Repeated random samplings of 400 quadrats from the data produced statistically indistinguishable results.

**BCI**						
**Growth of small focal trees vs:**	Df	Sum Sq	Mean Sq	F-value	Pr(>F)	Signif
Annular tree basal areas	6	747	124.5	128.8	<2e-16	***
Topography	3	207	69.1	71.4	<2e-16	***
Autocorrelations	396	3375	8.5	8.8	<2e-16	***
Basal areas x Topography	18	12	0.66	0.68	0.84	
Basal areas x Autocorrelations	2289	2481	1.08	1.12	4.5e-05	***
Residuals	212586	205579	0.97			
**Sinharaja**						
**Growth of small focal trees vs:**	Df	Sum Sq	Mean Sq	F-value	Pr(>F)	Signif
Annular tree basal areas	6	290	48.4	50.54	<2e-16	***
Topography	4	77	19.2	20.05	<2e-16	***
Autocorrelations	395	6873	17.4	18.17	<2e-16	***
Basal areas x Topography	24	32	1.32	1.38	0.103	
Basal areas x Autocorrelations	2314	2657	1.15	1.20	1.4e-10	***
Residuals	123518	118301	0.96			

*** indicates significance at the 0.001 level.

doi:10.1371/journal.pone.0156913.t001

the relationships between focal tree diameter and annular tree aggregation, recruitment and mortality, are all highly significant. The analyses also show, as expected, highly significant relationships between the response variables and topography, and between the response variables and autocorrelations among focal trees that share the same quadrat.

Table 2. Aggregation of annular trees vs diameters of focal conspecifics, plot topography, and autocorrelations between the focal trees that share each 20 x 20 m quadrat. Data from second census interval only.

**BCI**						
**Aggregation of annular conspeciics vs:**	Df	Sum Sq	Mean Sq	F-value	Pr(>F)	Signif
Focal tree diameters	3	6577	2192.5	763.6	<2e-16	***
Topography	3	116	38.8	13.5	8.19e-09	***
Autocorrelations	396	24695	62.4	21.7	<2e-16	***
Diameters x Topography	9	140	15.5	15.4	1.90e-07	***
Diameters x Autocorrelations	1187	7840	6.6	2.30	<2e-16	***
Residuals	57970	166446	2.9			
**Sinharaja**						
**Aggregation of annular conspecifics vs:**	Df	Sum Sq	Mean Sq	F-value	Pr(>F)	Signif
Focal tree diameters	3	75911	25304	1429.3	<2e-16	***
Topography	4	27440	6860	387.5	<2e-16	***
Autocorrelations	395	641195	1623	91.7	<2e-16	***
Diameters x Topography	12	1765	147	8.31	6.5e-16	***
Diameters x Autocorrelations	1184	59756	50	2.85	<2e-16	***
Residuals	91389	1617926	18			

Significance as in Table 1.

doi:10.1371/journal.pone.0156913.t002

Table 3. Recruitment of annular trees vs diameters of focal conspecifics, plot topography, and autocorrelations between the focal trees that share each 20 x 20 m quadrat.

**BCI**						
**Recruitment of annular conspecifics vs:**	Df	Sum Sq	Mean Sq	F-value	Pr(>F)	Signif
Focal tree diameters	3	1083	360.9	2039	<2e-16	***
Topography	3	12	4.0	22.84	1.01e-14	***
Autocorrelations	396	1848	4.7	26.4	<2e-16	***
Diameters x Topography	9	15	1.7	9.4	1.66e-14	***
Diameters x Autocorrelations	1188	652	0.5	3.1	<2e-16	***
Residuals	338412	59896	0.20			
**Sinharaja**						
**Recruitment of annular conspecifics vs:**	Df	Sum Sq	Mean Sq	F-value	Pr(>F)	Signif
Focal tree diameters	3	272	90.6	448.4	<2e-16	***
Topography	4	227	56.9	281.4	<2e-16	***
Autocorrelations	395	2465	6.24	30.9	<2e-16	***
Diameters x Topography	12	8	0.67	3.3	7.9e-05	***
Diameters x Autocorrelations	1184	374	0.32	1.6	<2e-16	***
Residuals	192518	38903	0.20			

Significance as in Table 1.

doi:10.1371/journal.pone.0156913.t003

Although most of the tests yield highly significant F-values, their precise level of significance cannot be determined from these ANOVA tests. With the exception of growth rates, the response variables are not normally distributed, and all the response variables show heteroskedasticity. However, it is possible to draw quantitative conclusions about the relative sizes of the fractions of variances of the response variables that are explained by the factors, and those that are explained by the pairwise interactions between the factors. In general, the interactions

Table 4. Mortality of annular trees vs diameters of focal conspecifics, plot topography, and autocorrelations between the focal trees that share each 20 x 20 m quadrat.

**BCI**						
**Mortality of annular conspecifics vs:**	Df	Sum Sq	Mean Sq	F-value	Pr(>F)	Signif
Focal tree diameters	3	695	231.6	1005.7	<2e-16	***
Topography	3	4	1.37	5.97	0.00046	***
Autocorrelations	396	2258	5.70	24.8	<2e-16	***
Diameters x Topography	9	12	1.39	6.02	1.72e-08	***
Diameters x Autocorrelations	1188	766	0.65	2.80	<2e-16	***
Residuals	338412	77924	0.23			
**Sinharaja**						
**Mortality of annular conspecifics vs:**	Df	Sum Sq	Mean Sq	F-value	Pr(>F)	Signif
Focal tree diameters	3	545	181.7	289.6	<2e-16	***
Topography	4	1183	295.8	471.4	<2e-16	***
Autocorrelations	395	21581	54.6	87.1	<2e-16	***
Diameters x Topography	12	43	3.62	5.78	4.32e-10	***
Diameters x Autocorrelations	1184	1448	1.22	1.95	<2e-16	***
Residuals	192518	120796	0.63			

Significance as in Table 1.

doi:10.1371/journal.pone.0156913.t004

explain substantially smaller fractions of the variance than the factors themselves. In almost all cases, the mean squares and F-values for these interactions are less than one percent of those for the variance components themselves. The magnitude of the NDD and PDD relationships revealed by the EAA analyses is unlikely to be affected substantially by these interactions.

Discussion

We are able to draw a number of conclusions that have ecological and evolutionary implications from the EAA and ANOVA analyses presented in this paper.

1) Positive and negative density-dependent effects are influenced by tree size

Both the negative density-dependent effects of annular trees on focal tree growth, and the positive within-species density-dependent facilitation mechanisms that enhance focal tree growth, are strongly dependent on focal tree size and summed annular tree basal area. If the summed basal areas of annular trees exceed focal tree basal area there is a negative effect on focal tree growth, and this negative effect increases with increasing difference between focal and annular trees. In contrast, the positive effect on the growth of the largest focal trees was greatest when the focal trees were surrounded by a large summed basal area of annular trees.

2) Positive and negative effects have different sources

If between-species density-dependent facilitation is the cause of the positive effect on large focal tree growth, it would be expected to be stronger in heterospecific than in conspecific interactions. Such a pattern of facilitation is not seen, so if it is present its effects must be smaller than the combined negative effect on growth rate of Janzen-Connell and niche-complementarity and thus not detectable. Instead, the growth rate of large focal trees is increased most strongly in the presence of a smaller summed basal area of annular conspecifics, suggesting a possible role for positive within-species density-dependent facilitation mechanisms on focal tree growth.

One testable hypothesis to explain this positive effect is that there may be an accumulation of beneficial host-specific symbionts such as mycorrhizae around large trees [60]. Another possibility is that the healthiest and fastest-growing large trees are those that by chance are in regions that have accumulated unusually small numbers of pathogens, so that they are more likely to be surrounded by conspecifics than more slowly-growing large trees. The extent and causes of this positive growth effect merit further investigation.

3) Tree distributions change with tree size

As focal trees survive and grow in size, the amounts of annular tree aggregation, recruitment and mortality around these survivors alter significantly in size. Figs 10–12 and Figs E and F of S1 File show the significance of these changes. A striking feature of these data is how quickly these measures begin to alter as focal trees increase in size. In most cases, the patterns are clearly different around small focal trees of 10–20 mm dbh and slightly larger focal trees of 20–50 mm dbh. The patterns continue to alter as focal trees increase in size.

These observations agree with observations of higher nonrandom mortality among the smallest trees in a Queensland, Australia forest plot [42]. Our results show the extent to which the surroundings of small and large trees in these plots are different, and show that similar differences are also detectable across large phylogenetic distances between focal and annular trees.

4) Subdivisions of plot data share properties

Analysis of forest plot data that has been divided into subgroups of species with different properties shows that size-, distance- and density-dependent effects are widely shared across these subgroups. Figs E and F of S1 File show that when species are subdivided into common, intermediate and rare, or into subgroups that have high, intermediate and low CVs of tree diameters, all these subgroups of species tend to exhibit similar properties when analyzed by the EAA method. These findings show that the NDD effects are widely distributed across species with different properties.

Our results from common, intermediate and rare species depart from those of earlier studies that have concentrated on the properties of seedlings. Comita and co-workers [61] found that seedlings of rare species at BCI showed higher mortality than seedlings of common ones when they were near conspecifics. They found, in contrast to the current study, that mortality was not significantly affected when the seedlings were near heterospecific trees. Using data from a wide range of forests plots, Johnson and co-workers [62] found similar results. Mangan et al. [11] found that seedlings of rare species showed stronger NDD effects than those of common species in experiments designed to test Janzen-Connell effects. Our data, which do not show this effect, are drawn only from trees that are larger than seedlings, and the data from our common, intermediate and rare species are the results of pooling information from a number of species. Application of the EAA method to seedling data, including pooling of species to increase the probability of detecting small effects, may help to quantify how the interactions between seedlings and their environment differ from those of larger trees.

5) Distantly related trees influence each other

The evolution of even distantly related species in these forest ecosystems continues to be influenced by significant non-random interactions between them. The sums of squares presented in Tables 1–4 show that the size-, density- and distance-related effects that have been explored in this study, many of which have been examined in other studies of the plots, explain much less than one percent of the total variances of focal tree size and growth. Nonetheless, even though these effects are small, they are highly significant and most of them remain significant across large phylogenetic distances between focal and annular trees. The phylogenetic persistence of these interactions indicates the presence of underlying ecological processes that contribute to the present-day dynamics of the forest communities. It seems possible that these interactions can also play continuing roles in the adaptive radiation of these tree species.

Part of the persistence over large phylogenetic distances may result from the fact that some fungal pathogens have been shown to be widely shared among all species in tropical forest ecosystems [63]. However, general niche-complementarity effects [15, 16], resulting from depletion of physical resources that are shared by even distantly-related species, could also contribute to this phylogenetic persistence.

The phylogenetic patterns that we have examined in these two forests are fully consistent with the strong phylogeny-associated effects of plant diseases that have been detected in a northern California grassland [35]. These authors found that the effects of diseases on plants were most pronounced when the plants were surrounded by conspecifics or closely related species. They also found that plant species introduced into a plot already heavily populated with closely-related species were more severely affected by disease than those that were introduced into a plot with fewer closely-related species.

Tree species that share an ecosystem can continue to influence each other's evolution long after their gene pools have become separated. Although rates of speciation vary greatly [64], prezygotic isolating mechanisms can accumulate rapidly in tropical ecosystems during

speciation. Female tropical sparrows respond differentially to differences in songs of males in populations separated by as little as 25 km [65]. Molecular data indicate that there is strong genetic isolation between rapidly evolving tropical tree and bromeliad species, even when there are ample opportunities for hybridization [66–68].

In the FDPs examined here, only closely-related pairs of species are likely to be able to exchange genes through introgression. The mean time back to a last common ancestor is 136.5 Ma at BCI and 139.8 Ma at Sinharaja, so that the majority of species pairs in these plots are expected to be genetically isolated—though of course the occasional horizontal gene transfer event can bridge even the largest phylogenetic gulf between species. Despite their genetic isolation, the properties of the most distantly related annular species show the same overall pattern of relationships with focal tree size and growth as do conspecifics—though the strength of these relationships is far weaker. Regardless of whether the sources of these interactions are shared physical resources, pathogens, herbivores or seed-predators, adaptive radiations among even distantly-related tree species that share the same ecosystem are able to continue, long after their gene pools have separated.

The conspecific interactions examined here constitute only a small part of the total variance in the demographic properties of the trees in the plots (Tables 1–4). This fraction drops further when species are phylogenetically separated. Even in these cases, the pooled data from one or a few five-year census intervals are sufficient to reveal details of these interactions. Over the millions of years during which these species-rich forests have existed, there must be a substantial cumulative effect of even such weak interactions on post-speciation adaptive radiation.

6) Divergence in resource use may result from interactions between distantly related species

We suggest an alternative to the notion that unpredictable tree neighborhoods should select for convergence in resource use. The possibility of convergence was first suggested by [69] and later elaborated by [70] and [71]. [71] modeled the process by which convergence in resource use could occur through adaptive evolution by dispersal-limited species with unpredictable neighbors. However, shared enemies (i.e., parasites and pathogens) are transmitted among individuals and over time they will increase in numbers on or within hosts. They differ in their properties, and apply different selective pressures to their hosts, than shared limiting resources that are depleted through exploitative competition. Shared enemies should favor divergent phenotypes, at all levels of negative consequences from infection and virulence that are experienced by the shared hosts. In other words, since fitness costs can accrue to hosts at all levels of infection and virulence, for two hosts that share a pathogen, selection should favor phenotypic divergence in both hosts even if they are distantly related and even if the levels of infection or virulence experienced from their shared pathogen differ between the hosts. Evidence for pronounced divergence between species in anti-herbivore traits, contrasted with smaller divergences in traits that are not related to herbivory, have been found at an Amazon forest site by [72].

We expect that application of the EAA method to other tropical, subtropical and temperate forests will provide further information about whether such persistent evolutionary pressures leading to evolutionary divergence are a universal feature of complex ecosystems.

Supporting Information

S1 File. Text A, Text B, Figs A-F.
(DOCX)

Acknowledgments

We gratefully acknowledge assistance from Cam Webb in the phylogenetic analysis, for the advice of Andreas Huth, Livio Fenga and Rick Condit, and for help in data analysis from Jerry Greenberg, Ron Hawkins and Paul Rodriguez of the San Diego Supercomputer Center. The detailed and constructive comments by reviewers of earlier versions of the manuscript were of great assistance in refining both the data analysis and its presentation. The BCI forest dynamics research project is supported by the National Science Foundation, the Center for Tropical Forest Science, the Smithsonian Tropical Research Institute, the John D. and Catherine T. MacArthur Foundation, the Mellon Foundation, the Small World Institute Fund, and numerous private individuals, and through the hard work of over 100 people from 10 countries over the past three decades. We thank the University of Peradeniya, Postgraduate Institute of Science and the Forest Department of Sri Lanka for administrative support, and the field staff who contributed to the censuses of the Sinharaja plot. These plot projects are part of the Center for Tropical Forest Science, a global network of large-scale demographic tree plots. BCI plot data are available from http://ctfs.arnarb.harvard.edu/webatlas/datasets/bci/. The Sinharaja Forest Monitoring Plot data belongs to the plot PIs and is managed by the CTFS-ForestGEO network. Data are available under request at http://ctfs.si.edu/Public/plotdataaccess/SiteDescription.php?plotname=sinharaja&typedata=tree. Thorsten Wiegand and Ruwan Punchi-Manage acknowledge funding from ERC advanced grant 233066-SPATIODIVERSITY. Programs for EAA analysis are available from C. Wills, cwills@ucsd.edu

Author Contributions

Conceived and designed the experiments: CW KH TW RP GG DE WK CVSG IAUNG. Performed the experiments: RP DE WK SH CVSG IAUNG. Analyzed the data: CW KH TW DE WK. Contributed reagents/materials/analysis tools: CW KH TW DE WK SH. Wrote the paper: CW KH TW RP GG DE WK SH.

References

1. Losos E, Leigh EG. Tropical forest diversity and dynamism: findings from a large-scale plot network. Chicago: University of Chicago Press; 2004.
2. Anderson-Teixeira KJ, Davies SJ, Bennett AC, Gonzalez-Akre EB, Muller-Landau HC, Joseph Wright S, et al. CTFS-ForestGEO: a worldwide network monitoring forests in an era of global change. Glob Change Biol. 2014; 21:529–49. doi: 10.1111/gcb.12712
3. Lebrija-Trejos E, Wright SJ, Hernández A, Reich PB. Does relatedness matter? Phylogenetic density dependent survival of seedlings in a tropical forest. Ecology. 2014;in press.
4. Comita LS, Queenborough SA, Murphy SJ, Eck JL, Xu KY, Krishnadas M, et al. Testing predictions of the Janzen-Connell hypothesis: a meta-analysis of experimental evidence for distance- and density-dependent seed and seedling survival. Journal of Ecology. 2014; 102(4):845–56. doi: 10.1111/1365-2745.12232 WOS:000338027500003. PMID: 25253908
5. Marhaver KL, Vermeij MJA, Rohwer F, Sandin SA. Janzen-Connell effects in a broadcast-spawning Caribbean coral: distance-dependent survival of larvae and settlers. Ecology. 2013; 94(1):146–60. WOS:000316186900017. PMID: 23600249
6. Janzen DH. Herbivores and the number of tree species in tropical forests. Am Nat. 1970; 104:501–29.
7. Connell JH. On the role of natural enemies in preventing competitive exclusion in some marine animals and in rain forest trees. In: Den Boer PJ, Gradwell G, editors. Dynamics of Populations. New York: PUDOC; 1971. p. 298–312.
8. Terborgh J. Enemies Maintain Hyperdiverse Tropical Forests. The American Naturalist. 2012; 179 (3):303–14. doi: 10.1086/664183 PMID: 22322219
9. Wright SJ. Plant diversity in tropical forests: a review of mechanisms of species coexistence. Oecologia. 2002; 130(1):1–14. doi: 10.1007/s004420100809 WOS:000173032500001.

10. Packer A, Clay K. Development of negative feedback during successive growth cycles of black cherry. Proceedings of the Royal Society B: Biological Sciences. 2004; 271(1536):317–24. doi: 10.1098/rspb.2003.2583 PMID: 15058444

11. Mangan SA, Schnitzer SA, Herre EA, Mack KML, Valencia MC, Sanchez EI, et al. Negative plant–soil feedback predicts tree-species relative abundance in a tropical forest. Nature. 2010; 466(7307):752–5. doi: 10.1038/nature09273 PMID: 20581819

12. Bagchi R, Gallery RE, Gripenberg S, Gurr SJ, Narayan L, Addis CE, et al. Pathogens and insect herbivores drive rainforest plant diversity and composition. Nature. 2014; 506(7486):85–8. doi: 10.1038/nature12911 http://www.nature.com/nature/journal/v506/n7486/abs/nature12911.html#supplementary-information. PMID: 24463522

13. Uriarte M, Condit R, Canham CD, Hubbell SP. A spatially explicit model of sapling growth in a tropical forest: does the identity of neighbours matter? Journal of Ecology. 2004; 92(2):348–60. doi: 10.1111/j.0022-0477.2004.00867.x WOS:000220382500015.

14. Uriarte M, Hubbell SP, John R, Condit R, Canham CD. Neighbourhood effects on sapling growth and survival in a neotropical forest and the ecological-equivalence hypothesis. In: Burslem DFRP, Pinard MA, Hartley SE, editors. Biotic interactions in the tropics: their role in the maintenance of species diversity Cambridge: Cambridge University Press; 2005. p. 89–106.

15. Shelly TE. Ecological comparisons of robber fly species (Diptera, Asilidae) coexisting in a neotropical forest. Oecologia. 1985; 67(1):57–70. doi: 10.1007/bf00378452 WOS:A1985ANM6000010.

16. Hector A, Schmid B, Beierkuhnlein C, Caldeira MC, Diemer M, Dimitrakopoulos PG, et al. Plant diversity and productivity experiments in European grasslands. Science. 1999; 286(5442):1123–7. doi: 10.1126/science.286.5442.1123 WOS:000083534200033. PMID: 10550043

17. Tilman D, Isbell F, Cowles JM. Biodiversity and Ecosystem Functioning. Annual Review of Ecology, Evolution, and Systematics. 2014; 45(1):471–93. doi: 10.1146/annurev-ecolsys-120213-091917

18. Bruno JF, Stachowicz JJ, Bertness MD. Inclusion of facilitation into ecological theory. Trends Ecol Evol. 2003; 18(3):119–25. doi: 10.1016/S0169-5347(02)00045-9

19. Wills C, Condit R, Foster RÄ, Hubbell SÄ. Strong density- and diversity-related effects help to maintain tree species diversity in a neotropical,Äâforest. Proceedings of the National Academy of Sciences. 1997; 94(4):1252–7.

20. Peters HA. Neighbour-regulated mortality: the influence of positive and negative density dependence on tree populations in species-rich tropical forests. Ecol Lett. 2003; 6(8):757–65. doi: 10.1046/j.1461-0248.2003.00492.x WOS:000184689900013.

21. Wills C, Harms KE, Condit R, King D, Thompson J, He F, et al. Nonrandom Processes Maintain Diversity in Tropical Forests. Science. 2006; 311(5760):527–31. doi: 10.1126/science.1117715 PMID: 16439661

22. Comita LS, Aguilar Sn, Pérez R, Lao S, Hubbell SP. Patterns of woody plant species abundance and diversity in the seedling layer of a tropical forest. Journal of Vegetation Science. 2007; 18(2):163–74.

23. Paine CET, Beck H. Seed predation by neotropical rain forest mammals increases diversity in seedling recruitment. Ecology. 2007; 88(12):3076–87. doi: 10.1890/06-1835.1 PMID: 18229842

24. Paine CET, Norden N, Chave J, Forget P-M, Fortunel C, Dexter KG, et al. Phylogenetic density dependence and environmental filtering predict seedling mortality in a tropical forest. Ecol Lett. 2012; 15(1):34–41. doi: 10.1111/j.1461-0248.2011.01705.x PMID: 22004454

25. Terborgh J, Nunez-Iturri G, Pitman NCA, Valverde FHC, Alvarez P, Swamy V, et al. Tree recruitment in an empty forest. Ecology. 2008; 89(6):1757–68. doi: 10.1890/07-0479.1 PMID: 18589539

26. Velho N, Isvaran K, Datta A. Rodent seed predation: effects on seed survival, recruitment, abundance, and dispersion of bird-dispersed tropical trees. Oecologia. 2012; 169(4):995–1004. doi: 10.1007/s00442-012-2252-9 PMID: 22327614

27. Bagchi R, Swinfield T, Gallery RE, Lewis OT, Gripenberg S, Narayan L, et al. Testing the Janzen-Connell mechanism: pathogens cause overcompensating density dependence in a tropical tree. Ecol Lett. 2010; 13(10):1262–9. doi: 10.1111/j.1461-0248.2010.01520.x PMID: 20718845

28. Bell T, Freckleton RP, Lewis OT. Plant pathogens drive density-dependent seedling mortality in a tropical tree. Ecol Lett. 2006; 9(5):569–74. doi: 10.1111/j.1461-0248.2006.00905.x PMID: 16643302

29. Yamazaki M, Iwamoto S, Seiwa K. Distance- and density-dependent seedling mortality caused by several diseases in eight tree species co-occurring in a temperate forest. Plant Ecology. 2009; 201(1):181–96. doi: 10.1007/s11258-008-9531-x

30. HilleRisLambers J, Clark JS, Beckage B. Density-dependent mortality and the latitudinal gradient in species diversity. Nature. 2002; 417(6890):732–5. doi: 10.1038/nature00809 WOS:000176154700042. PMID: 12066182

31. Coomes DA, Allen RB. Effects of size, competition and altitude on tree growth. Journal of Ecology. 2007; 95(5):1084–97. doi: 10.1111/j.1365-2745.2007.01280.x WOS:000249166700018.

32. Johnson DJ, Bourg NA, Howe R, McShea WJ, Wolf A, Clay K. Conspecific negative density-dependent mortality and the structure of temperate forests. Ecology. 2014; 95(9):2493–503. WOS:000342340200012.

33. Fricke EC, Tewksbury JJ, Rogers HS. Multiple natural enemies cause distance-dependent mortality at the seed-to-seedling transition. Ecol Lett. 2014; 17(5):593–8. doi: 10.1111/ele.12261 WOS:000333568600008. PMID: 24589220

34. Gilbert GS, Webb CO. Phylogenetic signal in plant pathogen-host range. Proc Natl Acad Sci U S A. 2007; 104(12):4979–83. doi: 10.1073/pnas.0607968104 WOS:000245256700040. PMID: 17360396

35. Parker IM, Saunders M, Bontrager M, Weitz AP, Hendricks R, Magarey R, et al. Phylogenetic structure and host abundance drive disease pressure in communities. Nature. 2015; 520(7548):542–4. doi: 10.1038/nature14372 http://www.nature.com/nature/journal/v520/n7548/abs/nature14372.html#supplementary-information. PMID: 25903634

36. Zhu K, Woodall CW, Monteiro JVD, Clark JS. Prevalence and strength of density-dependent tree recruitment. Ecology. 2015; 96(9):2319–27. doi: 10.1890/14-1780.1 PMID: 26594690

37. Liu X, Liang M, Etienne RS, Wang Y, Staehelin C, Yu S. Experimental evidence for a phylogenetic Janzen–Connell effect in a subtropical forest. Ecol Lett. 2012; 15(2):111–8. doi: 10.1111/j.1461-0248.2011.01715.x PMID: 22082078

38. Zhu Y, Comita LS, Hubbell SP, Ma K. Conspecific and phylogenetic density-dependent survival differs across life stages in a tropical forest. Journal of Ecology. 2015:

39. Chen Y, Wright SJ, Muller-Landau HC, Hubbell SP, Wang Y, Yu S. Positive effects of neighborhood complementarity on tree growth in a Neotropical forest. Ecology. 2016; 97(3):776–85. CCC:000373565900022. PMID: 27197403

40. Harms KE, Wright SJ, Calderon O, Hernandez A, Herre EA. Pervasive density-dependent recruitment enhances seedling diversity in a tropical forest. Nature. 2000; 404(6777):493–5. doi: 10.1038/35006630 WOS:000086257700047. PMID: 10761916

41. Lebrija-Trejos E, Wright SJ, Hernandez A, Reich PB. Does relatedness matter? Phylogenetic density-dependent survival of seedlings in a tropical forest. Ecology. 2014; 95(4):940–51. doi: 10.1890/13-0623.1 WOS:000334573600014. PMID: 24933813

42. Green PT, Harms KE, Connell JH. Nonrandom, diversifying processes are disproportionately strong in the smallest size classes of a tropical forest. Proceedings of the National Academy of Sciences. 2014; 111:18649–54. doi: 10.1073/pnas.1321892112

43. Condit R. Tropical forest census plots: methods and results from Barro Colorado Island, Panama, and a comparison with other plots. New York: Springer; 1998.

44. Hubbell SP, Foster RB, O'Brien ST, Harms KE, Condit R, Wechsler B, et al. Light-gap disturbances, recruitment limitation, and tree diversity in a neotropical forest. Science. 1999; 283(5401):554–7. doi: 10.1126/science.283.5401.554 WOS:000078203300042. PMID: 9915706

45. Ediriweera S, Singhakumara BMP, Ashton MS. Variation in canopy structure, light and soil nutrition across elevation of a Sri Lankan tropical rain forest. Forest Ecology and Management. 2008; 256(6):1339–49. doi: 10.1016/j.foreco.2008.06.035 WOS:000259762700013.

46. Kress WJ, Erickson DL, Jones FA, Swenson NG, Perez R, Sanjur O, et al. Plant DNA barcodes and a community phylogeny of a tropical forest dynamics plot in Panama. Proc Natl Acad Sci U S A. 2009; 106(44):18621–6. doi: 10.1073/pnas.0909820106 WOS:000271429800039. PMID: 19841276

47. Magallon S, Castillo A. Angiosperm diversification through time. American Journal of Botany. 2009; 96(1):349–65. doi: 10.3732/ajb.0800060 WOS:000262537900022. PMID: 21628193

48. Webb CO, Ackerly DD, Kembel SW. Phylocom: software for the analysis of phylogenetic community structure and trait evolution. Bioinformatics. 2008; 24(18):2098–100. doi: 10.1093/bioinformatics/btn358 WOS:000258959600020. PMID: 18678590

49. Wiegand T, Moloney KA. Rings, Circles, and Null-Models for Point Pattern Analysis in Ecology. Oikos. 2004; 104(2):209–29.

50. Condit R, Ashton PS, Baker P, Bunyavejchewin S, Gunatilleke S, Gunatilleke N, et al. Spatial patterns in the distribution of tropical tree species. Science. 2000; 288(5470):1414–8. doi: 10.1126/science.288.5470.1414 WOS:000087270900048. PMID: 10827950

51. Canham CD, Uriarte M. Analysis Of Neighborhood Dynamics Of Forest Ecosystems Using Likelihood Methods And Modeling. Ecol Appl. 2006; 16(1):62–73. doi: 10.1890/04-0657 PMID: 16705961

52. Diggle PJ. Statistical Analysis of Spatial Point Patterns. 2nd. Edition ed. London: Arnold; 2003.

53. Illian J, Penttinen A, Stoyan H, Stoyan D. Statistical Analysis and Modelling of Spatial Point Patterns. Chichester, UK: Wiley; 2008.

54. Kanagaraj R, Wiegand T, Comita LS, Huth A. Tropical tree species assemblages in topographical habitats change in time and with life stage. Journal of Ecology. 2011; 99(6):1441–52. doi: 10.1111/j.1365-2745.2011.01878.x WOS:000296425000015.

55. Punchi-Manage R, Getzin S, Wiegand T, Kanagaraj R, Gunatilleke CVS, Gunatilleke I, et al. Effects of topography on structuring local species assemblages in a Sri Lankan mixed dipterocarp forest. Journal of Ecology. 2013; 101(1):149–60. doi: 10.1111/1365-2745.12017 WOS:000317922200017.

56. Condit R, Hubbell SP, Foster RB. Recruitment near conspecific adults and the maintenance of tree and shrub diversity in a neotropical forest. Am Nat. 1992; 140(2):261–86. doi: 10.1086/285412 WOS: A1992JE84900004. PMID: 19426059

57. Metz MR, Sousa WP, Valencia R. Widespread density-dependent seedling mortality promotes species coexistence in a highly diverse Amazonian rain forest. Ecology. 2010; 91(12):3675–85. doi: 10.1890/08-2323.1 PMID: 21302838

58. Gunatilleke CVS, Gunatilleke I, Esufali S, Harms KE, Ashton PMS, Burslem D, et al. Species-habitat associations in a Sri Lankan dipterocarp forest. Journal of Tropical Ecology. 2006; 22:371–84. doi: 10.1017/s0266467406003282 WOS:000239587500002.

59. Harms KE, Condit R, Hubbell SP, Foster RB. Habitat associations of trees and shrubs in a 50-ha neotropical forest plot. Journal of Ecology. 2001; 89(6):947–59. WOS:000172926100004.

60. Smith SE, Read DJ, Harley JL. Mycorrhizal symbiosis. London: Academic Press; 1997.

61. Comita LS, Muller-Landau HC, Aguilar S, Hubbell SP. Asymmetric Density Dependence Shapes Species Abundances in a Tropical Tree Community. Science. 2010; 329(5989):330–2. doi: 10.1126/science.1190772 WOS:000279925900044. PMID: 20576853

62. Johnson DJ, Beaulieu WT, Bever JD, Clay K. Conspecific negative density dependence and forest diversity. Science. 2012; 336(6083):904–7. Epub 2012/05/19. doi: 10.1126/science.1220269 PMID: 22605774.

63. Gilbert GS, Magarey R, Suiter K, Webb CO. Evolutionary tools for phytosanitary risk analysis: phylogenetic signal as a predictor of host range of plant pests and pathogens. Evolutionary Applications. 2012; 5(8):869–78. doi: 10.1111/j.1752-4571.2012.00265.x WOS:000312808900009. PMID: 23346231

64. Rabosky DL, Matute DR. Macroevolutionary speciation rates are decoupled from the evolution of intrinsic reproductive isolation in Drosophila and birds. Proc Natl Acad Sci U S A. 2013; 110(38):15354–9. doi: 10.1073/pnas.1305529110 WOS:000324495300054. PMID: 24003144

65. Danner JE, Danner RM, Bonier F, Martin PR, Small TW, Moore IT. Female, but Not Male, Tropical Sparrows Respond More Strongly to the Local Song Dialect: Implications for Population Divergence. The American Naturalist. 2011; 178(1):53–63. doi: 10.1086/660283 PMID: 21670577

66. Palma-Silva C, Wendt T, Pinheiro F, Barbara T, Fay MF, Cozzolino S, et al. Sympatric bromeliad species (Pitcairnia spp.) facilitate tests of mechanisms involved in species cohesion and reproductive isolation in Neotropical inselbergs. Mol Ecol. 2011; 20(15):3185–201. doi: 10.1111/j.1365-294X.2011.05143.x WOS:000292963100010. PMID: 21672064

67. Surget-Groba Y, Kay KM. Restricted gene flow within and between rapidly diverging Neotropical plant species. Mol Ecol. 2013; 22(19):4931–42. doi: 10.1111/mec.12442 WOS:000324931300008. PMID: 23952304

68. Wendt T, Coser TS, Matallana G, Guilherme FAG. An apparent lack of prezygotic reproductive isolation among 42 sympatric species of Bromeliaceae in southeastern Brazil. Plant Systematics and Evolution. 2008; 275(1–2):31–41. doi: 10.1007/s00606-008-0054-7 WOS:000260220400003.

69. Connell JH. Diversity and the coevolution of competitors, or the ghost of competition past. Oikos. 1980; 35(2):131–8. doi: 10.2307/3544421 WOS:A1980KN89000002.

70. Hubbell SP, Foster RB. Biology chance and history and the structure of tropical rain forest tree communities1986. 314–30 p.

71. Hubbell SP. Neutral theory and the evolution of ecological equivalence. Ecology. 2006; 87(6):1387–98. doi: 10.1890/0012-9658(2006)87[1387:ntateo]2.0.co;2 WOS:000238658400006. PMID: 16869413

72. Endara M-J, Weinhold A, Cox JE, Wiggins NL, Coley PD, Kursar TA. Divergent evolution in antiherbivore defences within species complexes at a single Amazonian site. Journal of Ecology. 2015; 103(5):1107–18. doi: 10.1111/1365-2745.12431 WOS:000360216400002.
